# Vegetation Types and Plant Invasion Shape Pollination Interactions on a Tropical Island

**DOI:** 10.3390/plants15182845

**Published:** 2026-09-17

**Authors:** Laura Gómez-Devia, Christopher N. Kaiser-Bunbury, Anna Traveset, Alba Costa

**Affiliations:** 1 Mediterranean Institute for Advanced Studies (IMEDEA, CSIC-UIB), Mallorca, Illes Balears, 07190 Esporles, Spain; atraveset@csic.es (A.T.); a.costa@csic.es (A.C.); 2 Centre for Ecology and Conservation, Faculty of Environment, Science and Economy, University of Exeter, Cornwall Campus, Penryn TR10 9FE, UK; c.kaiser-bunbury@exeter.ac.uk

**Keywords:** ecological networks, species turnover, biological invasions, mutualisms, island ecology

## Abstract

Understanding how habitat transformation and non-native species shape plant–pollinator interactions is important for conserving and restoring oceanic islands, where both disturbances are widespread yet rarely examined together. Here, we investigated pollination networks across six contrasting vegetation types spanning a temporal gradient of invasion by non-native flowering plants on a tropical island. We recorded 5263 visits involving 327 unique plant–pollinator interactions over a three-month sampling period. We found that vegetation type and plant invasion intensity jointly structure different components of plant–pollinator networks at fine spatial scales. Pollinator assemblage composition differed more substantially across vegetation types than across months, although pollinators did not form distinct vegetation-type-specific assemblages. Plant–pollinator interactions also differed among vegetation types, primarily due to plant species turnover. In parallel, increasing invasion intensity was associated with pollinators visiting fewer native plant species, reducing the proportion of visits received by native plants and their importance to pollinators within the network. Moreover, non-native pollinators became more specialized with increasing invasion intensity. Flower abundance was negatively related to the contribution of both plant and pollinator species to nestedness, a structural network property associated with robustness, possibly reflecting altered pollinator visitation patterns associated with honeybee dominance. Overall, our findings highlight the importance of considering both vegetation heterogeneity and invasion intensity for understanding and conserving island plant–pollinator networks.

## 1. Introduction

Animal pollination constitutes an important ecosystem function, assisting the reproduction of the majority of flowering plant species (approx. 90%; [1]) and contributing to the maintenance of terrestrial biodiversity [2,3]. Understanding how plant–pollinator interactions respond to global change is therefore essential to safeguard ecosystem functioning [4,5]. Among the main drivers of global ecological change are habitat transformation (e.g., land-use change, habitat fragmentation and changes in vegetation composition) and the introduction of non-native species, both of which strongly influence pollination interactions [6]. Changes in vegetation composition and structure influence pollinator foraging behaviour through variation in floral resource density and diversity [7,8], whereas non-native species may divert pollinators from native plants, threatening their reproductive success [9,10]. Therefore, vegetation characteristics and non-native plants reshape pollination interaction networks by modifying the frequency and identity of plant–pollinator interactions through changes in floral resource availability, spatial distribution, and temporal overlap [11,12].

Oceanic islands provide particularly suitable ecosystems for studying how plant–pollinator interactions respond to habitat transformation and species introductions [11,12]. Most oceanic islands have undergone extensive habitat destruction, land exploitation, and mainland species introductions [13,14], generating a mosaic of plants of various ecological and evolutionary origins within relatively small areas. In addition, the isolation and small size of oceanic islands often result in relatively simple, species-poor plant–pollinator communities [15]. The reduced ecological complexity of oceanic islands facilitates detailed community-wide studies, often encompassing most or all interacting species and allowing the exploration of ecological responses to environmental change at finer scales [16,17,18].

Such community-wide characterization of species interactions can be effectively addressed through ecological network approaches, which provide a quantitative framework for assessing variation in plant–pollinator communities [19,20,21]. Species-level network metrics are particularly useful because they quantify and describe the roles of individual plants and pollinators within the wider community, including their connectivity, quantitative importance to interaction partners, and the degree of specialization [22,23,24]. These metrics offer complementary information on how species are embedded within ecological networks and allow detailed examination of shifts in interaction patterns across different ecological contexts, including changes in vegetation characteristics and invasion intensity, while distinguishing the responses of native and non-native plant and pollinator species [25,26].

On oceanic islands, habitat transformation and biological invasions often operate together, reshaping vegetation mosaics and altering the floral resources available to pollinators [27,28]. Recent paired comparisons between distinct vegetation types (e.g., forest and meadows, and forest and shrublands) indicate that microhabitat heterogeneity can shape pollination dynamics even over short spatial scales [29,30]. In addition, previous studies of island pollination networks have shown that the generalized nature of island pollination systems facilitates pollinator sharing between native and non-native plants, particularly when they have similar floral traits [17,31,32]. Yet, less is known about the joint influence of contrasting vegetation types and the abundance of non-native flowers on the foraging patterns of pollinators, plant–pollinator interactions and the differences between native and non-native species interaction patterns. Disentangling these effects on plant–pollinator networks is important for determining the role of species turnover, interaction rewiring among shared species, and the distribution of non-native floral resources in network dynamics.

Frégate Island, like many islands in the Seychelles archipelago, was severely transformed following the expansion of coconut plantations in the 19th century [33]. However, extensive restoration efforts over recent decades promoted the re-establishment of native forests through the reintroduction of endemic and native plant species [34,35]. As a result, Frégate Island contains a diverse mosaic of vegetation types within a relatively small area, including coastal vegetation, remnant coconut forests, native and mixed forests, gardens, and granitic glacis (bare rock), all characterized by distinct plant composition and vegetation structure. This makes Frégate Island a suitable natural system to examine how a mosaic of vegetation types and the variation in non-native flowering plants are associated with plant–pollinator interactions while minimizing broader environmental variation. These vegetation types are commonly found across the inner islands of the Seychelles archipelago, occurring in different proportions on each island.

Here, we surveyed plant–pollinator interactions across six vegetation types on Frégate Island and used community composition and ecological network analyses to examine how vegetation types and invasion intensity (measured as the proportion of open non-native flowers) shape pollinator assemblages, plant–pollinator interactions, and native and non-native species interaction patterns. Specifically, we addressed the following questions:

(1) Do pollinator foraging patterns differ among vegetation types? We anticipate that, given the small size of the island (c. 2 km^2^), the close proximity of vegetation types, and the absence of physical barriers, pollinator species will forage across multiple vegetation types rather than forming distinct vegetation-type-specific assemblages [29].
(2) Do plant–pollinator interaction patterns vary across vegetation types? Given that plant species identity and flower abundance influence pollination networks [7,36], we expect differences in plant species composition to correlate with differences in plant–pollinator interaction patterns across vegetation types.
(3) Does plant invasion intensity affect native and non-native plant and pollinator species differently? As non-native species can readily integrate into island pollination networks [9,31], we hypothesize increasing plant invasion intensity to be associated with a progressive shift in pollinator visitation from native to non-native flowers, negatively affecting native plants while benefiting non-native plants. In contrast, we anticipate native and non-native pollinators to respond similarly to plant invasion [37].

## 2. Results

Across all vegetation types, we recorded a total of 5263 pollinator visits belonging to 76 taxa, participating in 327 unique interactions with 41 plant species. A total of 174 (53%) of the observed interactions included at least one non-native species. The total number of native (20) and non-native (21) plant species was similar (full species list in Table S1). We recorded a higher proportion of native pollinators (39 species) than non-native ones (15 species) and 21 taxa could not be identified to species level (full species list in Table S2). Undetermined pollinators accounted for 6–17% of visits. To assess the influence of undetermined pollinators, we reran all models, including pollinator origin as a three-level factor (native, non-native, and undetermined). The results for the native and non-native pollinator categories remained qualitatively unchanged (Table S5). Most visits were by invertebrates (96%). Although Hymenoptera accounted for the largest proportion of invertebrate visits (56.8%), followed by Diptera (41.0%), Diptera exhibited greater taxonomic diversity, comprising 42 taxa compared with only 11 taxa in Hymenoptera. In addition, we recorded five vertebrate pollinators: two passerine birds (*Cinnyris dussumieri* and *Foudia sechellarum*), two reptiles (*Phelsuma astriata* and *Trachylepis seychellensis*) and the Seychelles fruit bat (*Pteropus seychellensis*). Together, vertebrates accounted for 4% of all visits, with birds contributing 3.37%, reptiles 0.57%, and the fruit bat recorded in a single visit (0.038%) (full species list in Table S2).

### 2.1. Spatial and Temporal Differences of Pollinator Assemblages

**Figure 1:**
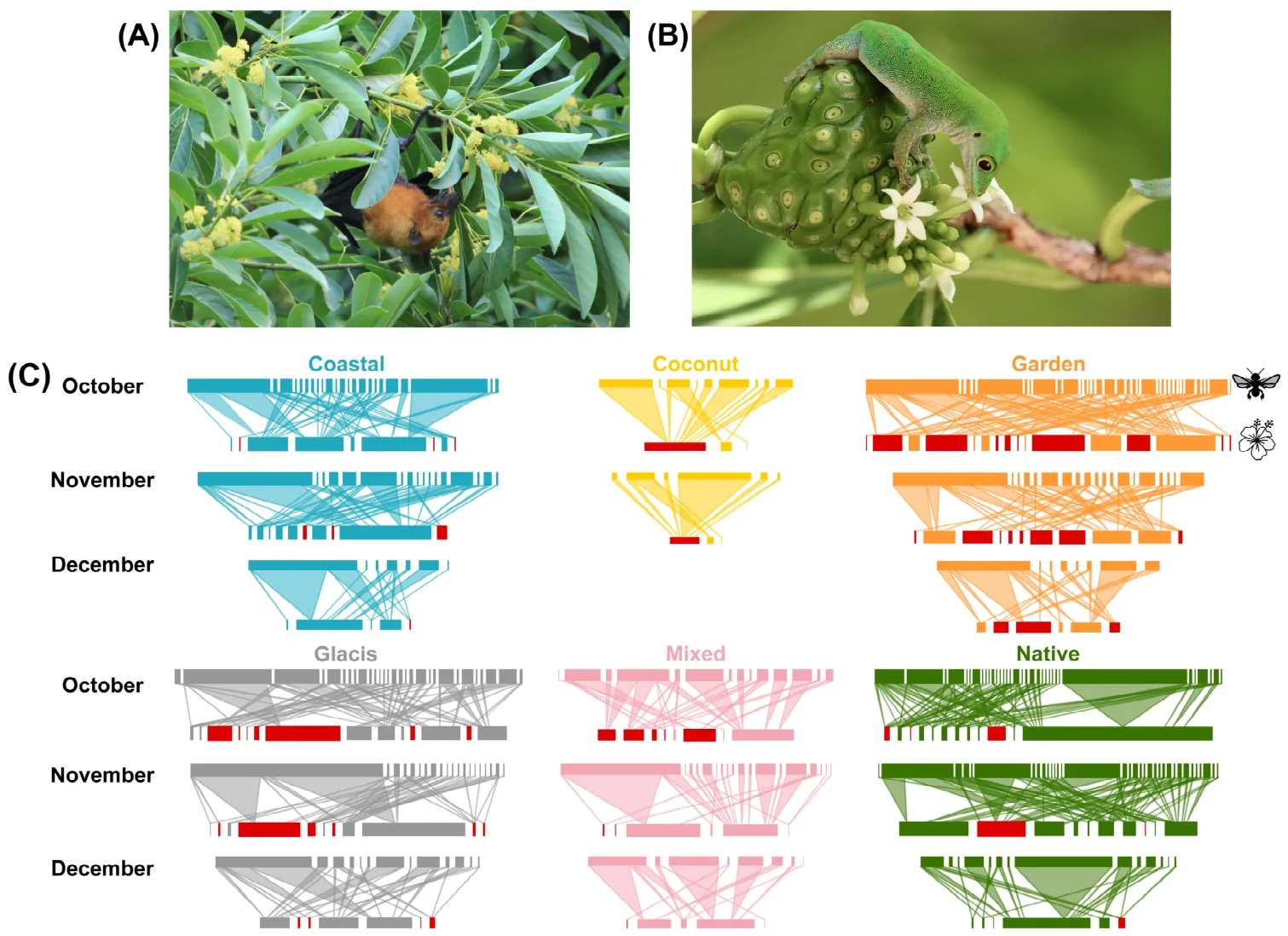
Frégate Island plant–pollinator interactions. (**A**) Endemic fruit bat (*Pteropus seychellensis*) pollinating non-native plant *Litsea glutinosa*. (**B**) Endemic day gecko (*Phelsuma astriata*) pollinating native plant *Morinda citrifolia.* (**C**) Pollination networks for each vegetation type and month. Top bars represent pollinators, and bottom bars plants. Dark red bars indicate non-native plant species. All 17 networks were drawn to the same scale.

Pollinator assemblages differed significantly among vegetation types (F_5,9_ = 2.527, R^2^ = 0.496, *p* = 0.0004) and among months (F_2,9_ = 1.834, R^2^ = 0.144, *p* = 0.017), with vegetation type explaining substantially more variation in pollinator assemblage composition than month. Multivariate dispersion did not differ among vegetation types (PERMDISP: F_5,11_ = 0.446, *p* = 0.658) or months (PERMDISP: F_2,14_ = 0.274, *p* = 0.439). In the NMDS ordination, pollinators did not cluster into distinct vegetation-type-specific assemblages, while pollinators from consecutive months tended to occur closer together in ordination space (Figure 1C).

### 2.2. Pollination Interaction Patterns Across Vegetation Types

On average, coconut forests exhibited the smallest networks and lowest interaction frequencies, whereas native forests had, on average, the largest networks, and coastal vegetation the highest mean interaction frequencies (Table 1; Figure 1C).

**Figure 2:**
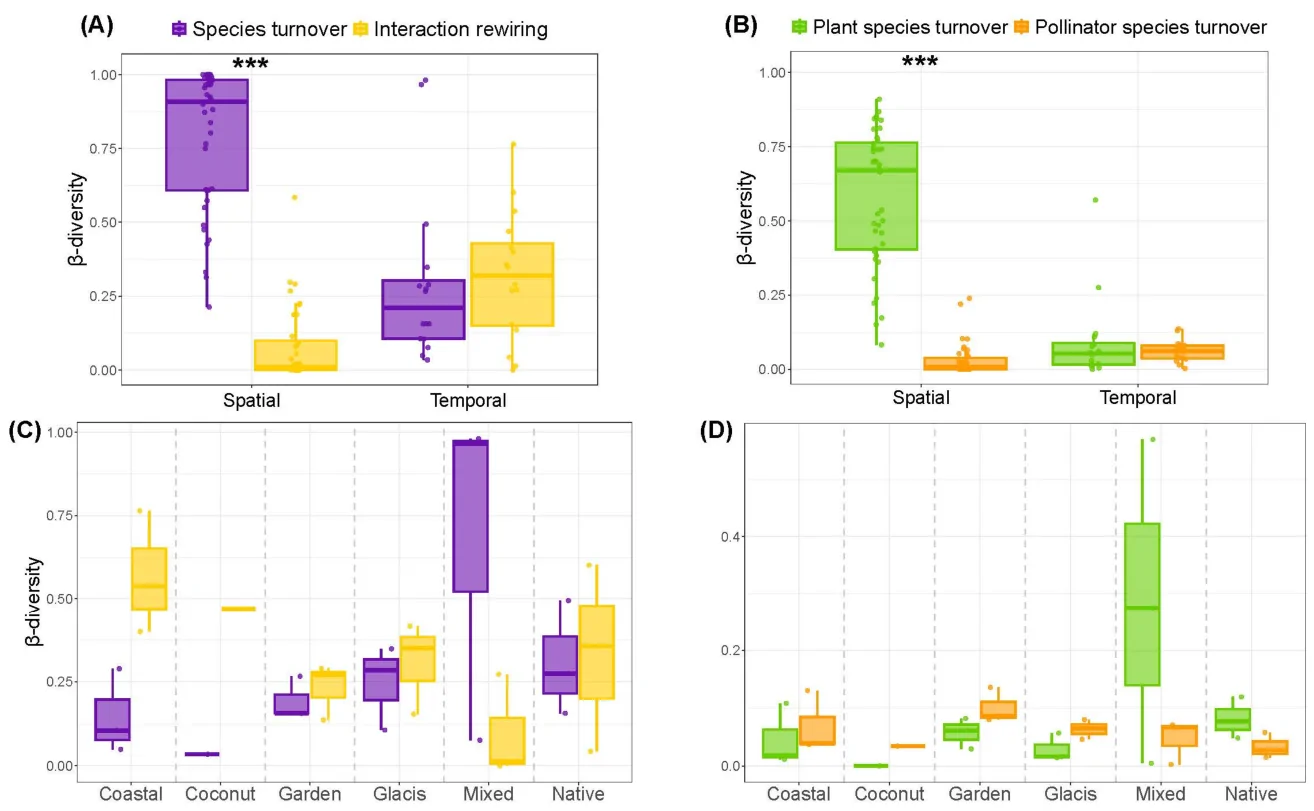
Comparison of network β-diversity components. (**A**) Spatial (n = 40; median paired difference = 0.905) and temporal (n = 16; median paired difference = −0.112) differences in species turnover (*β*_ST_) and interaction rewiring (*β*_OS_). (**B**) Spatial (n = 40; median paired difference = 0.647) and temporal (n = 16; median paired difference = −0.023). (**C**) Differences in species turnover and interaction rewiring within each vegetation type. (**D**) Differences in plant and pollinator species turnover within each vegetation type. Asterisks indicate significant differences (*** *p* < 0.0001).

Spatial differences in pollination interactions were driven primarily by species turnover (*β*_ST_: changes in plant and pollinator species composition) rather than by interaction rewiring (*β*_OS_: changes in interaction partners among co-occurring species) (V = 810, *p* < 0.0001, median paired difference = 0.905; Figure 2A). These patterns of species turnover were mainly driven by changes in plant species across vegetation types (V = 817, *p* < 0.0001, median paired difference = 0.647; Figure 2B). Species turnover and interaction rewiring contributed similarly to the temporal variation within vegetation types of pollination networks (V = 55, *p* = 0.528, median paired difference = −0.112). Likewise, plant and pollinator species turnover did not differ across the blooming season (V = 59, *p* = 0.669, median paired difference = −0.023).

A closer examination of the *β*-diversity components within vegetation types revealed contrasting patterns. In coastal and coconut forests, temporal changes in pollination networks were mainly driven by interaction rewiring, whereas species turnover played a smaller role. In contrast, species turnover was the dominant component in mixed forests. In gardens, glacis, and native forests, both components contributed similarly to *β*-diversity (Figure 2C). Species temporal turnover of plants and pollinators across vegetation types also varied. Pollinator turnover was slightly higher than plant turnover in coconut forests, gardens, and glacis, whereas plant turnover was higher in mixed and native forests. In coastal vegetation, plant and pollinator turnover were similar (Figure 2D).

**Figure 3:**
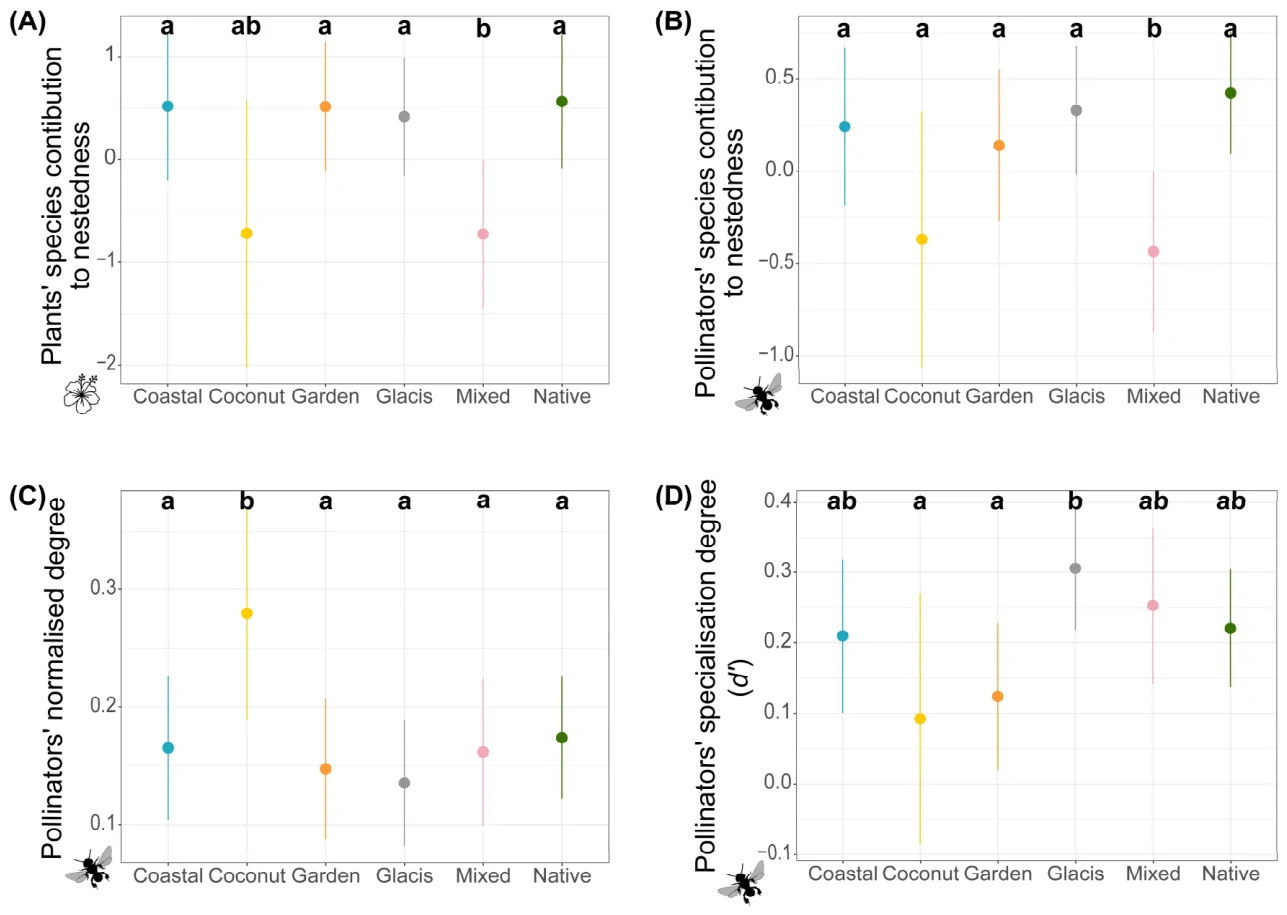
Species metrics that significantly differed between vegetation types. Points represent estimated marginal means (emmeans) from the fitted mixed-effects models, and error bars indicate 95% confidence intervals. Vegetation types with different lowercase letters differ significantly based on Holm-adjusted pairwise comparisons. The plant and pollinator models included n = 142 and n = 265 species-level observations, respectively. (**A**) Plant species contribution to nestedness. (**B**) Pollinator species contribution to nestedness. (**C**) Pollinators’ normalized degree. (**D**) Pollinators’ specialization degree (*d*’).

Species-level interaction patterns varied among vegetation types, with differences being more evident for pollinators than for plants. Mixed forests showed the lowest contribution to nestedness (CN) for both plants and pollinators; values were significantly different from all other vegetation types, except the coconut forests (*p* <0.01; CN; Figure 3A,B; Table S4a,b). Additionally, pollinators in coconut forests showed the highest normalized degree across all vegetation types, indicating greater connectivity than pollinators in all other vegetation types (ND: *p* <0.01; Figure 3C; Table S4b). Moreover, pollinators in glacis were the most selective, with significantly higher *d′* values than those in coconut forests and gardens (*p* <0.01; Figure 3D; Table S4b). The remaining species-level metrics, including plant and pollinator SS, plant ND, and plant *d*’, did not differ significantly among vegetation types.

### 2.3. Pollination Interaction Patterns Across Invasion Intensity

As plant invasion intensity increased, native plants showed a significant decline in normalized degree, indicating a lower proportion of interaction partners (ND: Est. =−0.0101, SE = 0.004, *p* = 0.018), and in species strength, indicating lower quantitative importance within the network (SS: Est. = −0.00876, SE = 0.0035, *p* = 0.013). Native plant contribution to nestedness also tended to decrease with invasion intensity, although this effect was not strong (CN: Est. = −0.0115, SE = 0.0061, *p* = 0.062) (Table S5; Figure S9). All other plant species-level metrics did not show a significant response to invasion intensity: *d*′ of both native and non-native plants, as well as ND, CN and SS of non-native plants.

Non-native pollinator specialization degree (*d*’) had a positive and significant relationship with invasion intensity (*d*’: Est. = 0.00282, SE = 0.00096, *p* = 0.004) (Table S6; Figure S10). All other native and non-native pollinator species-level metrics did not show a significant response to invasion intensity.

In addition, flower abundance was negatively associated with contribution to nestedness for both plants (CN: Est. = −0.0414, SE = 0.0166, *p* = 0.014) and pollinators (CN: Est. = − 0.00295, SE = 0.0106, *p* = 0.018). No other plant or pollinator species-level metrics were significantly influenced by flower abundance.

## 3. Discussion

The reduced ecological complexity of Frégate Island, together with its small spatial extent, allowed us to fully survey all flowering species during the sampling period and comprehensively characterize the plant–pollinator community and its interactions. This provided a detailed view of how vegetation heterogeneity and plant invasion shape pollination networks at fine spatial scales. While the overall direction of these patterns is consistent with previous studies, our results provide a detailed understanding of how the mosaic of vegetation types and invasion intensity jointly structures the plant–pollinator community through their effects on pollinator assemblages, interaction patterns, and species-level network structure.

The pollinator community across vegetation types did not form vegetation-type-specific assemblages. However, plant–pollinator interaction patterns varied, primarily through plant species turnover, indicating that changes in floral community composition were the main driver of differences in network structure, with interaction rewiring playing a comparatively smaller role. In parallel, increasing invasion intensity reduced the number of interacting partners (normalized degree) of native plants and their importance to pollinators within the network (species strength), while non-native pollinators were increasingly more selective in their interactions. In addition, higher flower abundance was associated with both plants and pollinator species contributing less to overall network nestedness.

### 3.1. Spatial and Temporal Differences of Pollinator Assemblages

Pollinator assemblages differed significantly across vegetation types and months, with vegetation type explaining substantially more variation in assemblage composition than month (vegetation type: R^2^ = 0.496; month: R^2^ = 0.144). The differences observed across vegetation types indicate that, despite the small spatial extent of the island and the absence of physical barriers, differences in the identity and availability of floral resources may have produced habitat-associated differences in pollinator foraging [29,30]. However, the NMDS ordination suggests that these differences reflect shifts in pollinator visitation patterns rather than discrete vegetation-type-specific assemblages. Thus, it suggests that pollinators used vegetation types differently while continuing to move among them and share floral resources, consistent with the high spatial connectivity of the island [38]. In turn, the temporal differences may reflect gradual seasonal changes in pollinator activity or floral resource availability during the main flowering period [39,40], consistent with the visual pattern of pollinators from consecutive months tending to occur closer together in the NMDS ordination.

### 3.2. Pollinator Interaction Patterns Across Vegetation Types

As expected, differences in pollination interactions between vegetation types were mainly driven by plant species turnover, indicating that variation in interaction patterns primarily reflected differences in the identity of floral resources available to pollinators, rather than interaction rewiring among co-occurring species. This suggests that spatial variation in pollination networks was largely determined by differences in the composition of the flowering plants, which changed the set of potential interaction partners available to pollinators across vegetation types. The comparatively low pollinator species turnover further supports the idea of a broadly shared pollinator pool across vegetation types. In contrast to this spatial pattern, species turnover and interaction rewiring contributed similarly to temporal variation within vegetation types, indicating that changes in pollination networks across months involved both changes in the species present and shifts in interactions among species persisting through time. The high variability in species turnover and interaction rewiring within vegetation types suggests that temporal changes in pollination networks may arise through different processes linked to the intrinsic characteristics of each vegetation type (i.e., vegetation structure and composition, canopy openness and microclimate).

The effects of the mosaic of vegetation types were restricted to specific components of the pollination interactions recorded and were not evident across all species-level metrics. In the mixed forest, both plant and pollinator species contributed significantly less to network nestedness than in most other vegetation types. The mixed forest also exhibited the highest species turnover, suggesting that the frequent replacement of interacting species may have reduced the persistence of shared interaction partners, thereby weakening the hierarchical interaction structure that underlies nestedness [41]. A similar pattern was observed in Puerto Rico, where forest pollination networks exhibited the highest species turnover [30].

The other differences in interaction patterns reported among vegetation types may be explained by the dominance of certain plant species that alter the distribution and accessibility of floral resources. In the coconut forest, pollinators exhibited the highest normalised degree, although this result should be interpreted with caution because of the particularly small network size of this vegetation type, which comprised only three plant species. The high normalized degree may therefore partly reflect the dominance of the invasive *C. nucifera* and the low diversity of floral resources available to pollinators. Under these conditions, the dominance of a single plant species may have concentrated pollinator interactions among a limited number of plant species [42]. In contrast, higher pollinator selectivity in the glacis may be associated with the dominance of the invasive shrub *Chrysobalanus icaco*, whose dense growth form may reduce pollinators’ access to alternative floral resources [14,25,43]. Moreover, *C. icaco* was visited predominantly by *A. mellifera* (93% of all visits), indicating that despite its high flower abundance, it supported relatively few pollinator species.

### 3.3. Species Interaction Structure Across Invasion Intensity

We found that invasion intensity negatively affected visits to native plant species. Our results, however, did not support the notion that non-native plant species benefited from invasion. As the proportion of non-native flowers increased, native plants received fewer visits from pollinators, reducing the connectivity and relative importance of native plants within pollination networks. As is common on oceanic islands, the native flora of Frégate Island is dominated by species with small, actinomorphic, white flowers bearing long exerted anthers [17], such as *Terminalia catappa*, *Calophyllum inophyllum*, *Mimusops sechellarum* and *Colubrina asiatica*. Similar floral traits are also present in many non-native species, including *Litsea glutinosa*, *Alstonia macrophylla*, *C. icaco* and *Cinnamomum verum*. Similarities in floral traits may have increased the likelihood of shared pollinators and therefore facilitated the integration of non-native plants into the island pollination networks [44,45]. The integration of non-native plants might have been further facilitated by the generalized and opportunistic behaviour of island pollinators. Indeed, the pollinators most frequently recorded visiting non-native plants were generalist species: the non-native honeybee (*Apis mellifera*, 34% of all visits to non-natives) and the native carpenter bee (*Xylocopa caffra*, 13% of all visits).

Furthermore, non-native pollinators became more specialized as plant invasion increased. Because *d*’ quantifies specialization relative to partner availability, this increase is unlikely to be explained solely by the greater abundance of non-native pollinators at more invaded sites. One possible explanation for such a pattern is that the increasing dominance of non-native flowers was associated with a lower diversity of floral resources available to pollinators, which may in turn have reduced pollinator functional redundancy [46]. The increase in pollinator specialization is consistent with findings from Mahé (Seychelles), where Kaiser-Bunbury et al. [37] reported increased specialization of different pollinator groups (e.g., bees, flies, and geckos) at sites dominated by non-native plants. Undetermined pollinator taxa may include both native and non-native species. However, our sensitivity analysis, which included pollinators of undetermined origin as a separate category, yielded qualitatively similar results for the native and non-native pollinator categories. This suggests that uncertainty in the identity of undetermined pollinators is unlikely to have influenced our interpretation of the effects of invasion intensity on the native and non-native pollinators identified.

Interestingly, flower abundance was negatively related to plant and pollinator contributions to nestedness. This pattern may be associated with the dominance of non-native *A. mellifera*, which can disproportionately exploit highly abundant floral resources and alter the visitation patterns of other pollinators. On Frégate Island, *A. mellifera* represented almost half of all interactions recorded during the three months sampled (43%), nearly four times as many as the second most frequent pollinator, *Rhodesiella rugosa* (12%). In addition, *A. mellifera* had the highest values for most species-level metrics across all vegetation types, further indicating the high activity levels across the island. Thus, the dominance of *A. mellifera* may be associated with altered visitation patterns on highly abundant flowering species, potentially reducing interaction diversity and contribution to nestedness. Similarly, Valido et al. [47] reported that honeybees disrupted the nested structure of wild-pollinator assemblages in the Canary Islands, leading to more modular networks. In line with this, fruit bats (*P. seychellensis*) are commonly observed visiting coconut flowers on North Island, another Seychelles island where honeybees are absent (LGD, pers. obs.). In contrast, on Frégate Island, coconut inflorescences were frequently surrounded by dense aggregations of honeybees, and no bat visits were recorded. Although these observations do not allow direct inference of competitive exclusion, they are consistent with the possibility that honeybee dominance may constrain visits from native pollinators. Given that honeybees are often less effective pollinators of native plants than native pollinators [48], these observations highlight the potential importance of managing honeybee populations [49].

Altogether, our study shows how non-native species can reorganize pollination networks within a heterogeneous oceanic island landscape, both through their floral abundance and through their vegetative structure, which influences the distribution and accessibility of floral resources. As non-native species reach high abundance and become dominant, they can displace native species [50,51]. This potentially leads not only to the homogenization of island communities but also to reduced diversity and changes in the structure of species interactions [7,10,52], similar to what was observed in the coconut forest and glacis vegetation types. In contrast, coastal and native forests, the vegetation types with the lowest invasion intensity, showed the highest interaction frequencies and largest networks, respectively. Therefore, restoring native species and limiting the spread of non-native ones could help maintain diverse floral resources and promote interaction diversity. Similar patterns were reported on Mahé Island (Seychelles), where the experimental removal of non-native shrubs increased pollinator as well as interaction diversity, flower visitation and the reproductive success of native plants [37]. Based on our results, restoration efforts could consider the prioritization of native species *Premna serratifolia* and *Terminalia catappa*, as these plants were visited by a high diversity of pollinator taxa (32 and 22 respectively). Moreover, we suggest prioritizing the control of the invasive shrub *C. icaco*, given that its abundant flowers were visited predominantly by *A. mellifera* and supported comparatively few pollinator taxa.

We must consider, however, that our study was conducted over a single flowering season and therefore does not capture interannual or seasonal variation in pollinator and flowering phenology. Future studies comparing interaction patterns between the dry and rainy seasons would help determine whether the differences in network structure observed among vegetation types remain consistent over time. Because vegetation types differed simultaneously in multiple habitat characteristics, such as canopy structure, vegetation composition, and associated microclimatic conditions, our study cannot disentangle the relative contribution of each of these components to the observed patterns. Incorporating direct measurements of canopy openness and microclimatic variables would allow us to identify the mechanisms underlying these differences. Moreover, our networks were also based on visitation frequency, which is a proxy for pollination: a high visitation rate does not necessarily translate into effective pollen transfer or plant reproductive success, and frequent visitors may contribute little to the pollination of the plants they visit. Complementing visitation data with measures of pollination effectiveness, such as single-visit pollen deposition or seed set, would allow future work to assess the functional consequences of the interaction shifts documented here. Beyond these approaches, controlled removal experiments involving non-native plant species could assess whether the effects of plant invasions on pollination networks differ among vegetation types. Finally, quantifying floral functional traits (e.g., morphology, colour, scent, and nectar rewards) along invasion gradients would allow us to identify the traits that facilitate pollinator sharing and the integration of non-native plants into pollination networks [53].

## 4. Methodology

### 4.1. Study Sites

**Figure 4:**
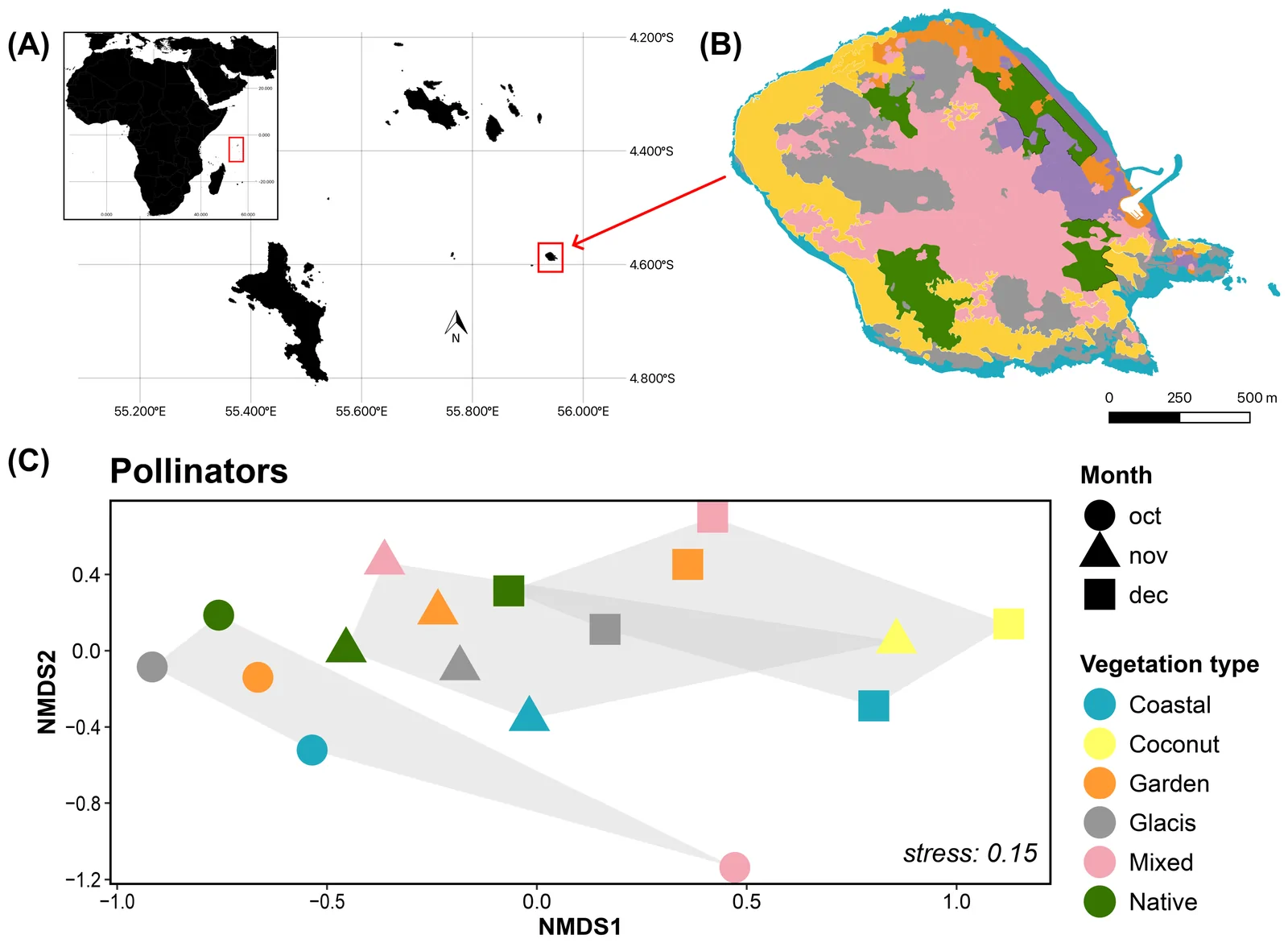
Study area, vegetation types, and pollinator species distribution on Frégate Island. (**A**) Geographic location of the Seychelles archipelago and Frégate Island. (**B**) Distribution of vegetation types across Frégate Island. (**C**) Non-metric multidimensional scaling (NMDS) of pollinator visitation patterns. Symbols indicate sampling month, and colours represent vegetation types. Grey polygons enclose the vegetation types sampled within each month.

Data was collected on Frégate Island (Indian Ocean 4°35′19′′ S, 55°56′55′′ E). Frégate Island is located 55 km away from Mahé (the main Seychelles island; Figure 4A) and has an area of 2.07 ${km}^{2}$, with the highest peak reaching 125 m a.s.l. The island is privately owned, with approximately one-third of it occupied by a small resort that accommodates between 100 and 150 people, depending on guest occupancy. The climate is tropical, characterized by a dry season from May to October (Southeast Monsoon) and a wet season from December to March (Northwest Monsoon). Temperatures range between 26 and 32 °C and humidity between 78 and 82% [54].

We identified vegetation types based on vegetation structure and plant species composition (Table 2, Figure 4B). Invasion intensity varied among vegetation types across the three sampling months and was therefore not used to classify them. Flower abundance was recorded for each vegetation type by counting all flowering individuals at the beginning of each month along fixed, 4 m wide linear transects. The number of transects per vegetation type was proportional to the total area occupied by each vegetation type on the island, to achieve comparable spatial sampling coverage across vegetation types. This resulted in two to five transects and a total sampled transect length of 150–500 m per vegetation type (Table 2). Transects were established once at the beginning of the study and resurveyed in the same locations throughout the entire sampling period. For species producing numerous flowers, including tall trees where complete counts were not feasible, flower abundance was estimated based on the visible proportion of the canopy and extrapolated according to tree size. Flower abundance was then expressed as the number of flowers per m^2^. We subsequently calculated invasion intensity as the percentage of open non-native flowers relative to the total number of open flowers. This measure was selected because our study focuses on plant–pollinator interactions, for which floral abundance and composition are the components of plant invasion that most directly influence pollinator foraging behaviour and interaction patterns [55,56].

### 4.2. Data Collection

We collected plant-pollinator interaction data during the main flowering season, from 1 October until 11 December 2023. We conducted focal observations on all plant species that were flowering within each vegetation type during the sampling period. For each plant species, during each month and in each vegetation type, we conducted six fixed focal observations of 30 min each, totalling three hours of observation, thereby standardizing sampling effort while capturing variation in pollinator activity throughout the day. We aimed to observe six different plant individuals, randomly selecting individuals with flowers in good condition while avoiding those with rotten or dry flowers. When fewer than six individuals were available, plant individuals were observed more than once to maintain a total observation time of three hours, with repeated observations conducted on different days and at different times of the day to minimize temporal clustering and associated sampling bias. Observations were conducted between 07:30 and 18:00 under sunny or partly cloudy conditions. Focal observations were systematically scheduled, with the order of observations across vegetation types randomized to ensure that each plant species was observed evenly throughout the day, including morning, midday, and afternoon observation periods. A pollinator visit was recorded only when the individual contacted the reproductive structures of the flower. Visitors were identified in the field when possible; however, most specimens were collected with an insect aspirator and preserved in ethanol for later identification by an expert taxonomist. Voucher specimens were deposited in the Mediterranean Institute for Advanced Studies (IMEDEA) insect collection.

### 4.3. Network Analysis

We built monthly quantitative plant-pollinator interaction networks for each vegetation type. Plant-pollinator interaction frequency was used as a measure of interaction strength. For each plant–pollinator pair, interaction frequency was calculated as the number of visits by pollinator *i* to plant *j* per observed flower per focal session, weighted by an independent community-level estimate of the flower abundance of plant species *j* (visits × flower^−1^ × h^−1^ × *j* flower abundance) [57]. Focal observations were pooled for each plant species within each vegetation type and month before calculating interaction frequencies and constructing the networks. Visitation rates were then weighted by flower abundance to account for differences in floral-resource availability, following the approach used in comparable island plant–pollinator network studies [31,37]. For each network, using the *bipartite* R-package (v2.24; [58]), we calculated four different species-level metrics for both plants and pollinators: normalized degree (ND), contribution to nestedness (CN), species strength (SS) and specialization degree (*d*’). ND quantifies the partners a species interacts with in relation to the total number of possible partners in the network, providing a measure of its connectivity within the network [24,59]. CN measures how much a species contributes to the nested structure in a network, where nestedness occurs when specialist species interact with subsets of the partners of generalist species [60]. Higher CN values identify species that contribute strongly to this hierarchical organization, a network structure associated with greater stability in mutualistic communities [61,62]. SS quantifies the summed dependence of a species’ partners, reflecting the relative importance of that species for its interaction partners [22]. Species specialization degree, *d*’, describes the selectiveness in partner use relative to partner availability, ranging from zero (generalist, or opportunistic) to one (specialist, selective) [46]. All metrics were calculated within networks constructed using the same standardized sampling protocol to reduce variation associated with sampling effort. We chose these four metrics because they provide complementary information on the frequency and diversity of species interactions, enabling us to characterize the structure of plant–pollinator networks and distinguish species-specific interaction patterns between native and non-native plant species [19,20].

### 4.4. Statistical Analysis

#### 4.4.1. Quantifying Spatial and Temporal Differences of Pollinator Assemblages

Using plant–pollinator interaction data obtained from the focal observations, we compared pollinator assemblages between vegetation types and sampling months. Focal observations were pooled by vegetation type and month, resulting in one pollinator assemblage per vegetation type per month. We constructed a Bray–Curtis dissimilarity matrix based on pollinator species identity and number of visits as a proxy for abundance (i.e., one visit = one individual). Differences in pollinator assemblage composition were tested using permutational multivariate analysis of variance (PERMANOVA), with vegetation type and sampling month included in the same additive model and their marginal effects tested while accounting for the other factor. We used 9999 permutations, restricting permutations within sampling month when testing the effect of vegetation type and within vegetation type when testing the effect of month. Because PERMANOVA can be sensitive to differences in within-group dispersion, we additionally assessed homogeneity of multivariate dispersion using PERMDISP, applying the corresponding restricted permutation schemes for vegetation type and month. Patterns were visualized using non-metric multidimensional scaling (NMDS). All analyses were performed in R using the R package vegan (v 2.7.5; [63]).

#### 4.4.2. Pollination Interaction Patterns Across Vegetation Types and Invasion Intensity

To assess changes in plant–pollinator interactions across vegetation types, we quantified interaction *β*–diversity among networks using Whittaker’s dissimilarity index [64]. Interaction *β*–diversity was partitioned into two components: (1) species turnover (*β*_ST_), which measures interaction differences due to species losses or gains between networks, and (2) interaction rewiring (*β*_OS_), which accounts for interaction differences resulting from partner switching among species shared between networks. Specifically, we used the *betalinkr* function from the *bipartite* R-package (v2.24; [58]), specifying the “commondenom” partitioning, which enables separate assessment of plant (*β*_S.L_) and pollinator (*β*_S. H_) species turnover [58,65]. We quantified interaction *β*–diversity spatially, by comparing networks between vegetation types, and temporally, by comparing monthly networks within each vegetation type [29]. We then compared β-diversity components (*β*_ST_ vs. *β*_OS_ and *β*_S.L_ vs. *β*_S.H_), derived from the same network pair, spatially and temporally using paired Wilcoxon signed-rank tests. Spatial (n = 40) and temporal (n = 16) sample sizes refer to pairwise network comparisons rather than independent ecological replicates, as individual networks can occur in multiple pairwise comparisons. We additionally calculated the median paired difference between β-diversity components as an effect-size measure.

To evaluate differences in species interaction patterns across vegetation types and to test whether native and non-native species responded differently to invasion intensity, we ran linear mixed models (LMMs) with Gaussian distribution. Plant and pollinator species metrics were set as response variables (ND, CN, SS and *d*’). To meet model assumptions, ND, SS and *d*’ were logarithmically transformed. For each model, fixed effects included vegetation type and the interaction between species origin (native vs. non-native) × invasion. The interaction term was included to explicitly test if the effect of invasion varied depending on species origin. Additionally, flower abundance was also included as a fixed effect, as resource availability is known to strongly influence species interactions [66]. Continuous explanatory variables were entered into the models on their original scales. Species identity and sampling month were included as random effects to account for repeated observations of species and temporal dependence among monthly observations. Month was excluded as a random effect from the models of plant and pollinator *d*′ and pollinator SS because its variance was estimated as zero and resulted in a singular fit. Model diagnostics were carried out with the DHARMa R-package (v.0.4.7; [67]). The final models showed no substantial deviations from normality or homoscedasticity (Table S3; Figure S1–S8). Differences across vegetation types were tested using post-hoc estimated marginal means (emmeans), applying the Holm correction for multiple testing and Kenward–Roger approximation for degrees of freedom [68]. All analyses were conducted in R (v4.4.3; R Core Team, 2025).

## Figures and Tables

**Table 1:** Summary of pollination networks in each vegetation type. Nat. = native species, Intr. = non-native species, Undet. = undetermined. Network size = total plant species × total pollinator species.

Vegetation Type	Month	Plant Species		Pollinator Species			Network Size			Interaction Frequency		
		Nat.	Intr.	Nat.	Int.	Undet.	Monthly	Mean	s.d	Monthly	Mean	s.d
Coastal	October	6	3	14	5	4	207	137.34	90.53	7.23	6.19	4.08
	November	7	3	12	4	1	170			1.68		
	December	4	1	6	1	0	35			9.64		
Coconut	November	2	1	8	0	1	27	22.5	6.36	0.54	0.42	0.17
	December	2	1	6	0	0	18			0.30		
Garden	October	6	10	20	7	7	544	267	253.04	2.29	1.63	0.64
	November	3	8	8	8	3	209			1.59		
	December	3	3	5	2	1	48			1.01		
Glacis	October	8	6	17	7	5	406	239	158.36	1.00	1.47	0.41
	November	5	6	13	6	1	220			1.68		
	December	3	4	8	5	6	91			1.74		
Mixed	October	3	5	9	1	4	112	86.67	40.46	0.38	1.60	1.5
	November	4	2	12	4	2	108			3.29		
	December	4	1	6	2	0	40			1.14		
Native	October	9	3	17	7	10	408	277.34	170.85	11.99	5.94	5.57
	November	8	2	22	9	3	340			4.50		
	December	5	1	8	5	1	84			1.32		

**Table 2:** Summary of Frégate Island vegetation types. Invasion level is expressed as the mean proportion (%) of non-native flowers ± standard deviation calculated across the three months sampled.

Vegetation Type	Size (ha)	N° Transects	Transect Length (m)	Invasion Level (%)	Floral Diversity	Characteristics
Coastal	1.03	2	150	21 ± 30	1.02 ± 0.53	Sandy beaches and rocky cliff areas with little or no shade, exposed to strong winds and predominantly composed of native shrubs (3–4 m tall), with *Scaevola taccada* as the dominant species.
Coconut	4.31	4	400	76 ± 26	0.9 ± 0.72	Dense, tall stands (~30 m) dominated by *Cocos nucifera*, mainly restricted to the periphery of the island.
Garden	0.88	3	150	67 ± 15	1.93 ± 0.26	Dominated by non-native ornamental plants, uniformly located next to the road and hotel villas, usually not very tall (~3 m) and producing little or no shade.
Glacis	3.92	4	350	42 ± 35	1.6 ± 0.86	Sparse vegetation cover, generally composed of short plants (3–4 m) with little or no shade and exposed to extreme temperatures. Contains a diversity of native and endemic plant species, such as *Euphorbia pyrifolia*, *Pyrostria bibracteata* and *Premna serratifolia*, but is also highly invaded by non-native *Chrysobalanus icaco.*
Mixed	4.88	5	500	41 ± 36	1.51 ± 0.09	Dense areas with very tall trees (10–40 m), comprising a mixture of non-native species, such as *Cinnamomum verum*, *Alstonia macrophylla* and *Anacardium occidentale*, and native species such as *P. serratifolia* and *Morinda citrifolia.*
Native	2.20	3	250	39 ± 10	1.59 ± 0.38	Restored areas dominated by replanted native tree species (e.g., *Calophyllum inophyllum*, *Mimusops sechellarum* and *Rothmannia annae*), with a sparse understory and tree heights reaching up to ~20 m.

## Data Availability

All raw data necessary to reproduce the analyses are provided as an Excel file in the Supplementary Material. Further inquiries can be directed to the corresponding author.

## Supplementary Materials

The following supporting information can be downloaded at https://www.mdpi.com/article/10.3390/plants15182845/s1: Figures S1–S8. DHARMa residual diagnostics for the species-level mixed-effects models: Figure S1: Plants’ normalized degree; Figure S2: Plants’ contribution to nestedness; Figure S3: Plants’ species strength; Figure S4: Plants’ specialization degree (d’); Figure S5: Pollinators’ normalized degree; Figure S6: Pollinators’ contribution to nestedness; Figure S7: Pollinators’ species strength; Figure S8: Pollinators’ specialization degree (d’); Figure S9: Predicted relationships between invasion intensity and plant species-level metrics for native and non-native plants; Figure S10: Predicted relationships between invasion intensity and pollinator species-level metrics for native and non-native pollinators; Table S1: Plant species list for all vegetation types; Table S2: Pollinator species list for all vegetation types; Table S3: Response variables and transformations used in Gaussian mixed-effects models evaluated with DHARMa residual diagnostics; Table S4: Species-level metrics for vegetation types pairwise comparisons. Post-hoc estimated marginal means (emmeans) test, applying Holm correction for multiple testing and Kenward–Roger approximation for degrees of freedom; Table S5: LMM statistical model results for plant species-level metrics; Table S6: LMM statistical model results for pollinator species-level metrics.
