# Supplementary material for: Vegetation Types and Plant Invasion Shape Pollination Interactions on a Tropical Island
Source: Plants (Basel). 2026 Sep 17;15(18):2845. doi: 10.3390/plants15182845 (PMC13611578; doi:10.3390/plants15182845)
Supplement: Supplementary file 1 [file plants-15-02845-s001.zip › plants-4441982-Supplementary Material.pdf]

Supplementary material : Vegetation types and plant invasion shape pollination interactions on a tropical island

Laura Gómez-Devia[1*], Christopher Kaiser-Bunbury[2], Anna Traveset[1] and Alba Costa[1]

[1]*Mediterranean Institute for Advanced Studies (IMEDEA, CSIC-UIB), Esporles, Mallorca Illes Balears Spain.*[2]*Centre for Ecology and Conservation, Faculty of Environment, Science and Economy, University of Exeter, Cornwall Campus, Penryn, UK*

Table S1. Plant species list for all vegetation types

Vegetation type	Plant family	Plant species	origin
Coastal	Acanthaceae	Asystasia gangetica	native
	Apocynaceae	Catharanthus roseus	introduced
	Rhamnaceae	Colubrina asiatica	native
	Boraginaceae	Cordia subcordata	native
	Boraginaceae	Heliotropium arboreum	native
	Hernandiaceae	Hernandia nymphaeifolia	native
	Convolvulaceae	Ipomoea obscura	introduced
	Convolvulaceae	Ipomoea pes-caprae	native
	Passifloraceae	Passiflora foetida	introduced
	Goodeniaceae	Scaevola taccada	native
	Verbenaceae	Stachytarpheta indica	introduced
Coconut	Arecaceae	Cocos nucifera	introduced
	Boraginaceae	Cordia subcordata	native
	Rubiaceae	Morinda citrifolia	native
Garden	Apocynaceae	Allamanda cathartica	introduced
	Anacardiaceae	Anacardium occidentale	introduced
	Acanthaceae	Asystasia gangetica	native
	Fabaceae	Bahuinia variegata	introduced
	Calophyllaceae	Calophyllum inophyllum	native
	Apocynaceae	Cerbera manghas	native
	Chrysobalanaceae	Chrysobalanus icaco	introduced
	Lauraceae	Cinnamomum verum	introduced
	Euphorbiaceae	Codiaeum variegatum	introduced
	Apocynaceae	Cryptostegia madagascariensis	introduced
	Malvaceae	Hibiscus tiliacaeus	native
	Euphorbiaceae	Jatropha integerrima	introduced
	Rubiaceae	Morinda citrifolia	native
	Apocynaceae	Plumeria obtusa	introduced
	Lamiaceae	Premna serratifolia	native
	Verbenaceae	Stachytarpheta indica	introduced
	Acanthaceae	Thunbergia affinis	introduced

Glacis	Fabaceae	Abrus precatorius	native
	Fabaceae	Adenanthera pavonina	native
	Apocynaceae	Alstonia macrophylla	introduced
	Anacardiaceae	Anacardium occidentale	introduced
	Acanthaceae	Asystasia gangetica	native
	Apocynaceae	Catharanthus roseus	introduced
	Chrysobalanaceae	Chrysobalanus icaco	introduced
	Lauraceae	Cinnamomum verum	introduced
	Euphorbiaceae	Euphorbia pyrifolia	native
	Ochnaceae	Ochna kirkii	introduced
	Lamiaceae	Premna serratifolia	native
	Rubiaceae	Pyrostria bibracteata	native
	Verbenaceae	Stachytarpheta indica	introduced
	Apocynaceae	Tabernaemontana coffeoides	native
	Fabaceae	Tephrosia noctiflora	introduced
	Combretaceae	Terminalia catappa	native
Mixed	Apocynaceae	Alstonia macrophylla	introduced
	Anacardiaceae	Anacardium occidentale	introduced
	Chrysobalanaceae	Chrysobalanus icaco	introduced
	Lauraceae	Cinnamomum verum	introduced
	Lauraceae	Litsea glutinosa	introduced
	Sapotaceae	Mimusops sechellarum	native
	Rubiaceae	Morinda citrifolia	native
	Lamiaceae	Premna serratifolia	native
	Myrtaceae	Psidium cattleanium	introduced
	Apocynaceae	Tabernaemontana coffeoides	native
	Combretaceae	Terminalia catappa	native
Native	Anacardiaceae	Anacardium occidentale	introduced
	Calophyllaceae	Calophyllum inophyllum	native
	Chrysobalanaceae	Chrysobalanus icaco	introduced
	Lauraceae	Litsea glutinosa	introduced
	Sapotaceae	Mimusops sechellarum	native
	Rubiaceae	Morinda citrifolia	native
	Apocynaceae	Ochrosia oppositifolia	native
	Lamiaceae	Premna serratifolia	native
	Rubiaceae	Pyrostria bibracteata	native
	Rubiaceae	Rothmannia annae	native
	Verbenaceae	Stachytarpheta indica	introduced
	Apocynaceae	Tabernaemontana coffeoides	native
	Combretaceae	Terminalia catappa	native

Table S2. Pollinator species list for all vegetation types

Vegetation type	Pollinators order	Pollinator species	Pollinators origin
	Hymenoptera	Apis mellifera	introduced
	Diptera	Cadrema pallida (Loew, 1866)	native
	Hymenoptera	Ceratina tabescens Cockerell, 1912	native
	Hymenoptera	Chalicodoma disjuncta	introduced
	Passeriformes	Cinnyris dussumieri	native
	Diptera	Desmometopa m-nigrum (Zetterstedt, 1848)	introduced
	Diptera	Diptera_sp3	undetermined
	Coleoptera	Exochomus laeviusculus	native
	Diptera	Forcipomyia sp.2	undetermined
	Diptera	Gaurax seychellensis Lamb, 1912	native
	Hymenoptera	Knemodynerus seychellensis (Dalla Torre, 1904)	native
	Undetermined	LEP G5 s1	undetermined
	Hymenoptera	Lasioglossum mahense	native
	Diptera	Limnophora exigua (Wiedemann, 1830)	introduced
	Diptera	Lobosciara bilotata Vilkamaa & Hippa, 1994	native
	Diptera	Lucilia papuensis Macquart, 1843	introduced
	Hymenoptera	Megachile seychellensis Cameron, 1907	native
	Diptera	Morellia insularis Pont, 2006	native
	Diptera	Musca domestica	introduced
	Diptera	Neavella albipectus (Bigot, 1859)	native
	Diptera	Paralimna (Paralimna) bicolor (Macquart, 1851)	native
	Squamata	Phelsuma astriata	native
	Diptera	Sapromyza nudiuscula Lamb, 1912	native
	Diptera	Sarcophaga (Afrothyrsocnema) seychellica (Verves, 1986)	native
	Diptera	Scaptodrosophila caliginosa (Lamb, 1914)	native
	Diptera	Scaptodrosophila finitima (Lamb, 1914)	native
	Squamata	Trachylepis seychellensis	native
	Lepidoptera	Utetheisa pulchelloides	introduced
	Hymenoptera	Xylocopa caffra	native
Coastal	Hemiptera	undetermined Psyllidae Gen.2 sp.1	undetermined
Coconut	Hymenoptera	Apis mellifera	introduced
	Diptera	Cadrema pallida (Loew, 1866)	native

	Passeriformes	Cinnyris dussumieri	native
	Passeriformes	Foudia sechellarum	native
	Hemiptera	Hemip_sp2	undetermined
	Hymenoptera	Lasioglossum mahense	native
	Diptera	Lobosciara bilotata Vilkamaa & Hippa, 1994	native
	Squamata	Phelsuma astriata	native
	Diptera	Scholastes vicarius	native
	Hymenoptera	Apis mellifera	introduced
	Diptera	Arcuator stigmaticus (Lamb, 1912)	native
	Diptera	Atherigona orientalis Schiner, 1868	introduced
	Diptera	Atrichopogon yamabukiensis Clastrier,1987	native
	Diptera	Cadrema pallida (Loew, 1866)	native
	Hymenoptera	Ceratina tabescens Cockerell, 1912	native
	Hymenoptera	Chalicodoma disjuncta	introduced
	Diptera	Chrysomya megacephala	introduced
	Passeriformes	Cinnyris dussumieri	native
	Diptera	Diptera_sp2	undetermined
	Diptera	Diptera_sp4	undetermined
	Diptera	Eristalinus seychellarum	native
	Hemiptera	HEM MIRI G5 s1*	undetermined
	Hymenoptera	Knemodynerus seychellensis (Dalla Torre, 1904)	native
	Hymenoptera	Lasioglossum mahense	native
	Diptera	Limnophora exigua (Wiedemann, 1830)	introduced
	Diptera	Lobosciara bilotata Vilkamaa & Hippa, 1994	native
	Diptera	Lonchaea minuta de Meijere, 1910	introduced
	Diptera	Lucilia papuensis Macquart, 1843	introduced
	Hymenoptera	Megachile seychellensis Cameron, 1907	native
	Diptera	Morellia insularis Pont, 2006	native
	Diptera	Musca domestica	introduced
	Squamata	Phelsuma astriata	native
	Diptera	Physiphora alceae (Preyssler, 1791)	introduced
	Diptera	Pseudogaurax sp. 1	undetermined
	Diptera	Scaptodrosophila finitima (Lamb, 1914)	native
	Diptera	Scholastes vicarius	native
	Squamata	Trachylepis seychellensis	native
	Hymenoptera	Xylocopa caffra	native
Garden	Orthoptera	Zarceus fallaciosus Bolívar, 1895	native

	Lepidoptera	brown moth (2 cm)	undetermined
	Undetermined	undetermined	undetermined
	Undetermined	undetermined Nitidulidae Gen.1 sp.1	native
	Hemiptera	undetermined Miridae Gen.9 sp.1	undetermined
	Undetermined	undetermined Muscidae Gen.1 sp.1	undetermined
	Undetermined	undetermined Scolytidae Gen.2 sp.1	native
	Undetermined	undetermined Sliferia	native
	Diptera	Aphanotrigonum subfasciella Collin, 1949	native
	Hymenoptera	Apis mellifera	introduced
	Diptera	Arcuator stigmaticus (Lamb, 1912)	native
	Hemiptera	Austroagallia caboverdensis (Lindberg, 1958)	introduced
	Diptera	Cadrema pallida (Loew, 1866)	native
	Hymenoptera	Ceratina tabescens Cockerell, 1912	native
	Hymenoptera	Chalicodoma disjuncta	introduced
	Diptera	Chrysomya megacephala	introduced
	Passeriformes	Cinnyris dussumieri	native
	Diptera	Culex pipiens Linnaeus, 1758	introduced
	Diptera	Dichaetomyia fasciculifera (Stein, 1910)	native
	Diptera	Diptera_sp1	undetermined
	Diptera	Diptera_sp2	undetermined
	Diptera	Diptera_sp4	undetermined
	Diptera	Eristalinus seychellarum	native
	Hemiptera	Hemip_sp1	undetermined
	Hymenoptera	Knemodynerus seychellensis (Dalla Torre, 1904)	native
	Hymenoptera	Lasioglossum mahense	native
	Diptera	Limnophora exigua (Wiedemann, 1830)	introduced
	Diptera	Lobosciara bilotata Vilkamaa & Hippa, 1994	native
	Diptera	Lonchaea minuta de Meijere, 1910	introduced
	Diptera	Lucilia papuensis Macquart, 1843	introduced
	Hymenoptera	Megachile seychellensis Cameron, 1907	native
	Diptera	Morellia insularis Pont, 2006	native
	Diptera	Musca albomaculata	native
	Diptera	Musca domestica	introduced
	Squamata	Phelsuma astriata	native
	Diptera	Rhodesiella (Rhodesiella) rugosa (Lamb, 1912)	native
Glacis	Diptera	Sarcophaga (Afrothyrsocnema) seychellica (Verves, 1986)	native

	Diptera	Scaptodrosophila finitima (Lamb, 1914)	native
	Diptera	Scholastes vicarius	native
	Squamata	Trachylepis seychellensis	native
	Lepidoptera	Trigonodes hypassia	native
	Hymenoptera	Xylocopa caffra	native
	Orthoptera	Zarceus fallaciosus Bolívar, 1895	native
	Undetermined	undetermined Cynipidae Gen. 1 sp. 1	undetermined
	Hemiptera	undetermined Miridae Gen.9 sp.1	undetermined
	Undetermined	undetermined Platygastridae Gen.3 sp.1	undetermined
	Undetermined	undetermined Sliferia	native
Mixed	Diptera	Anthomyia amoena (Macquart, 1851)	native
	Hymenoptera	Apis mellifera	introduced
	Diptera	Arcuator stigmaticus (Lamb, 1912)	native
	Diptera	Cadrema pallida (Loew, 1866)	native
	Diptera	Chaetonerius alluaudi (Giglio-Tos, 1895)	native
	Diptera	Chrysomya megacephala	introduced
	Passeriformes	Cinnyris dussumieri	native
	Diptera	Culex pipiens Linnaeus, 1758	introduced
	Diptera	Diptera_sp3	undetermined
	Diptera	Diptera_sp4	undetermined
	Diptera	Forcipomyia sp.1	undetermined
	Passeriformes	Foudia sechellarum	native
	Hymenoptera	Knemodynerus seychellensis (Dalla Torre, 1904)	native
	Hymenoptera	Lasioglossum mahense	native
	Diptera	Limnophora exigua (Wiedemann, 1830)	introduced
	Diptera	Lobosciara bilotata Vilkamaa & Hippa, 1994	native
	Diptera	Lucilia papuensis Macquart, 1843	introduced
	Diptera	Morellia insularis Pont, 2006	native
	Diptera	Musca domestica	introduced
	Squamata	Phelsuma astriata	native
	Diptera	Plagiostenopterina cyanosoma Hendel, 1912	native
	Chiroptera	Pteropus seychellensis	native
	Diptera	Rhodesiella (Rhodesiella) rugosa (Lamb, 1912)	native
	Diptera	Scaptodrosophila finitima (Lamb, 1914)	native
	Diptera	Scholastes vicarius	native
	Hymenoptera	Xylocopa caffra	native

	Orthoptera	Zarceus fallaciosus Bolívar, 1895	native
	Lepidoptera	brown moth (2 cm)	undetermined
	Undetermined	undetermined Chloropidae Gen.5 sp0.2	undetermined
	Undetermined	undetermined Muscidae Gen.1 sp.1	undetermined
	Diptera	Aphanotrigonum subfasciella Collin, 1949	native
	Hymenoptera	Apis mellifera	introduced
	Diptera	Arcuator stigmaticus (Lamb, 1912)	native
	Diptera	Atherigona orientalis Schiner, 1868	introduced
	Diptera	Cadrema pallida (Loew, 1866)	native
	Diptera	Chaetonerius alluaudi (Giglio-Tos, 1895)	native
	Hymenoptera	Chalicodoma disjuncta	introduced
	Diptera	Chrysomya megacephala	introduced
	Passeriformes	Cinnyris dussumieri	native
	Diptera	Culex pipiens Linnaeus, 1758	introduced
	Diptera	Diptera_sp3	undetermined
	Diptera	Diptera_sp4	undetermined
	Diptera	Eristalinus seychellarum	native
	Lepidoptera	Euproctis fraterna	native
	Coleoptera	Exochomus laeviusculus	native
	Diptera	Forcipomyia sp.2	undetermined
	Hemiptera	HEM LYGA G3 s1*	undetermined
	Hemiptera	Hyalopeplus rama (Kirby, 1891)	introduced
	Hymenoptera	Knemodynerus seychellensis (Dalla Torre, 1904)	native
	Hymenoptera	Lasioglossum mahense	native
	Diptera	Limnophora exigua (Wiedemann, 1830)	introduced
	Diptera	Lobosciara bilotata Vilkamaa & Hippa, 1994	native
	Diptera	Lonchaea minuta de Meijere, 1910	introduced
	Diptera	Lucilia papuensis Macquart, 1843	introduced
	Hymenoptera	Megachile seychellensis Cameron, 1907	native
	Diptera	Morellia insularis Pont, 2006	native
	Diptera	Musca albomaculata	native
	Diptera	Musca domestica	introduced
	Diptera	Neavella albipectus (Bigot, 1859)	native
	Squamata	Phelsuma astriata	native
	Diptera	Physiphora alceae (Preyssler, 1791)	introduced
Native	Diptera	Plagiostenopterina cyanosoma Hendel, 1912	native

Diptera	Pseudogaurax sp. 1	undetermined
Diptera	Rhinia apicalis (Wiedemann, 1830)	native
Diptera	Rhodesiella (Rhodesiella) rugosa (Lamb, 1912)	native
Diptera	Sarcophaga (Afrothyrsocnema) seychellica (Verves, 1986)	native
Diptera	Scaptodrosophila finitima (Lamb, 1914)	native
Diptera	Scholastes vicarius	native
Squamata	Trachylepis seychellensis	native
Hymenoptera	Xylocopa caffra	native
Lepidoptera	brown moth (2 cm)	undetermined
Hymenoptera	undetermined Agaonidae Gen.1 sp.1	undetermined
Undetermined	undetermined Chloropidae Gen.5 sp.1	undetermined
Undetermined	undetermined Epidapus sp.1	undetermined
Hemiptera	undetermined Miridae Gen.9 sp.1	undetermined
Hemiptera	undetermined Miridae nymph	undetermined

DHARMa residual diagnostics

Model:

Species metric ~ vegetation type + (species origin * invasion intensity) + flower abundance + (1|species)

Table S3. Response variables and transformations used in Gaussian mixed-effects models evaluated with DHARMa residual diagnostics.

Figure	Taxonomic group	Species metric	Response transformation
Fig. S1	Plants	Normalized degree	log-transformed
Fig. S2	Plants	Contribution to nestedness	None
Fig. S3	Plants	Species strength	log-transformed
Fig. S4	Plants	Specialisation degree (d')	log-transformed
Fig. S5	Pollinators	Normalized degree	log-transformed
Fig. S6	Pollinators	Contribution to nestedness	None
Fig. S7	Pollinators	Species strength	log-transformed

Figure	Taxonomic group	Species metric	Response transformation
Fig. S8	Pollinators	Specialisation degree (d')	log-transformed

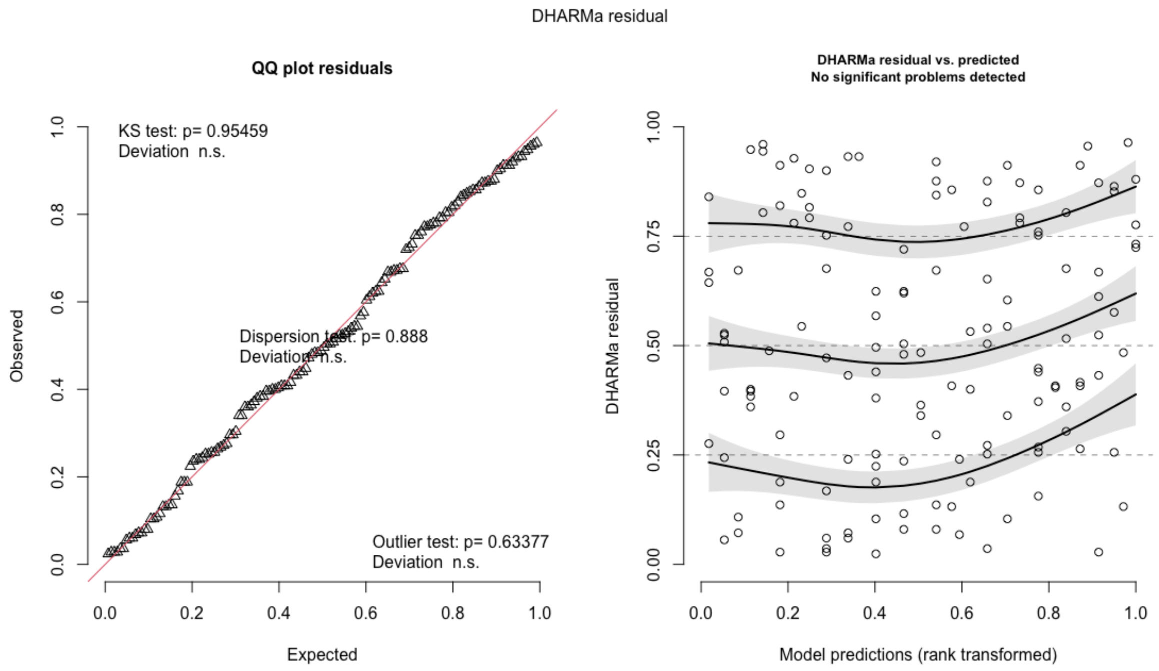

Figure S1. Plants normalised degree

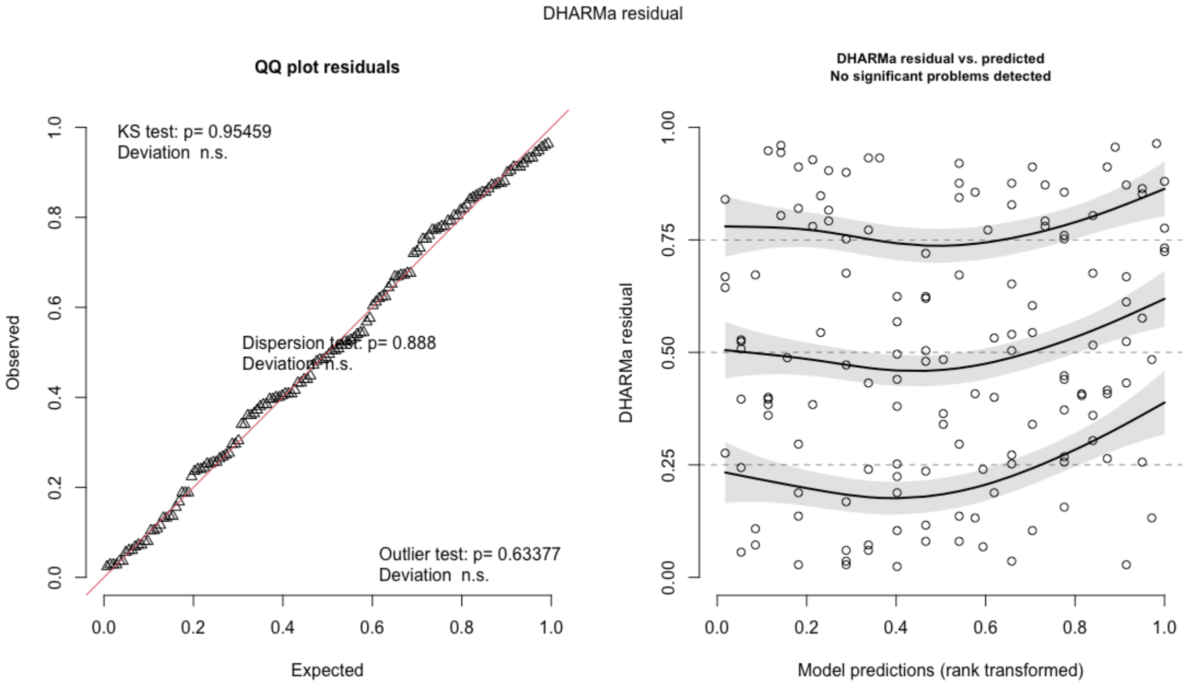

Figure S2. Plants contribution to nestedness

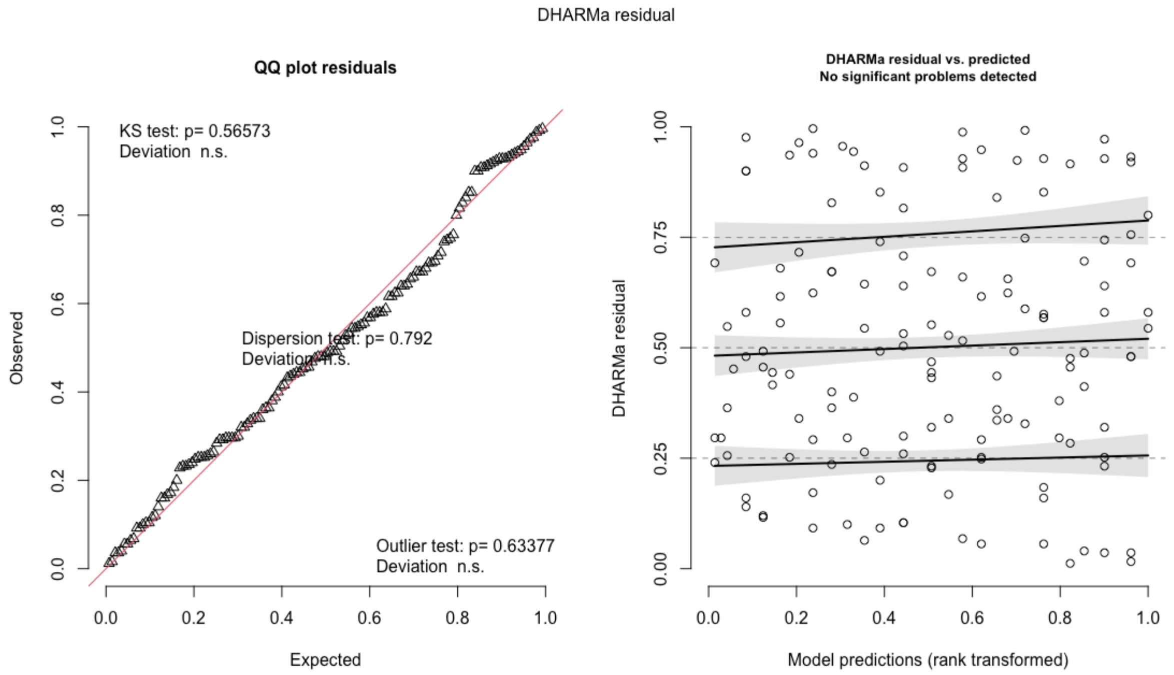

Figure S3. Plants species strength

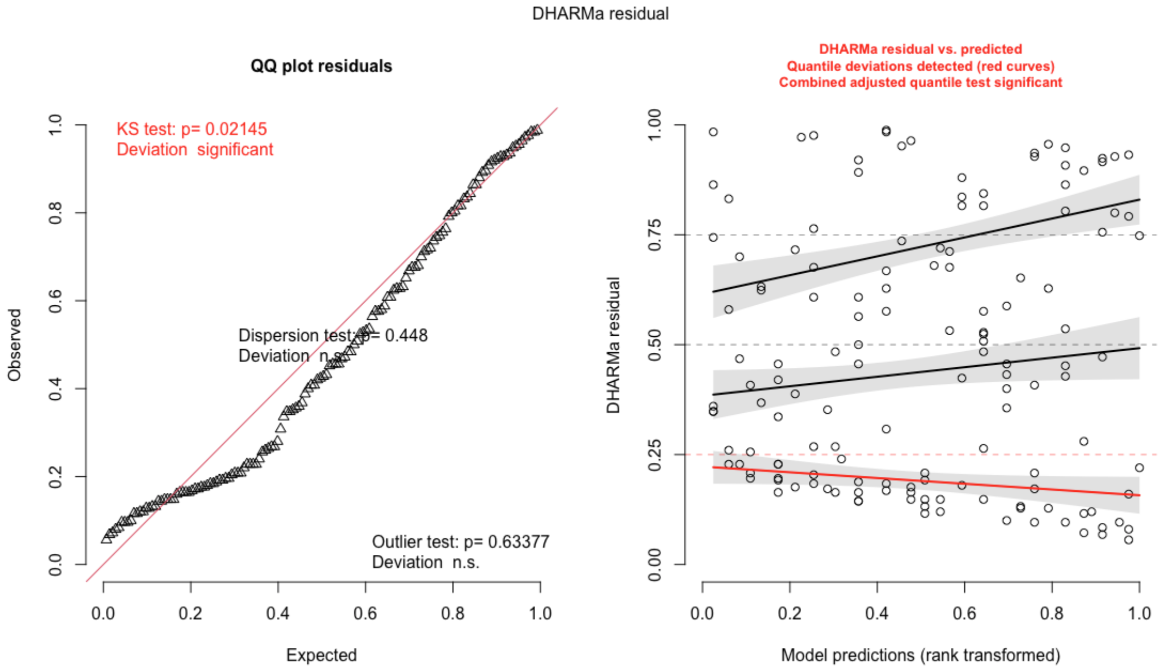

Figure S4. Plants specialization degree (*d*')

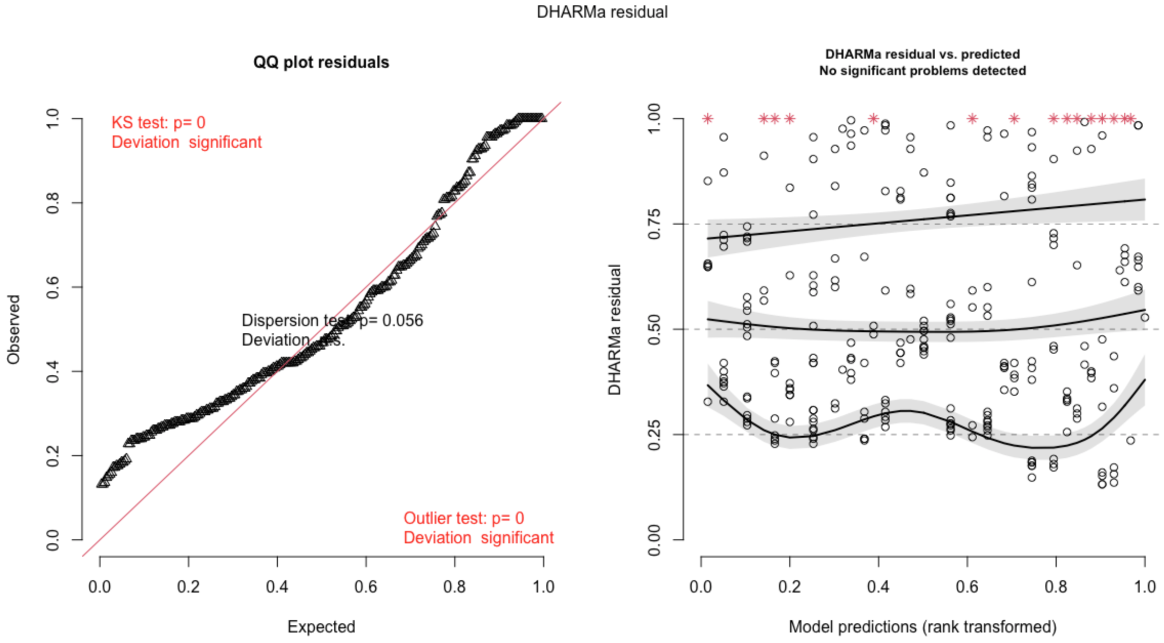

Figure S5. Pollinators normalised degree

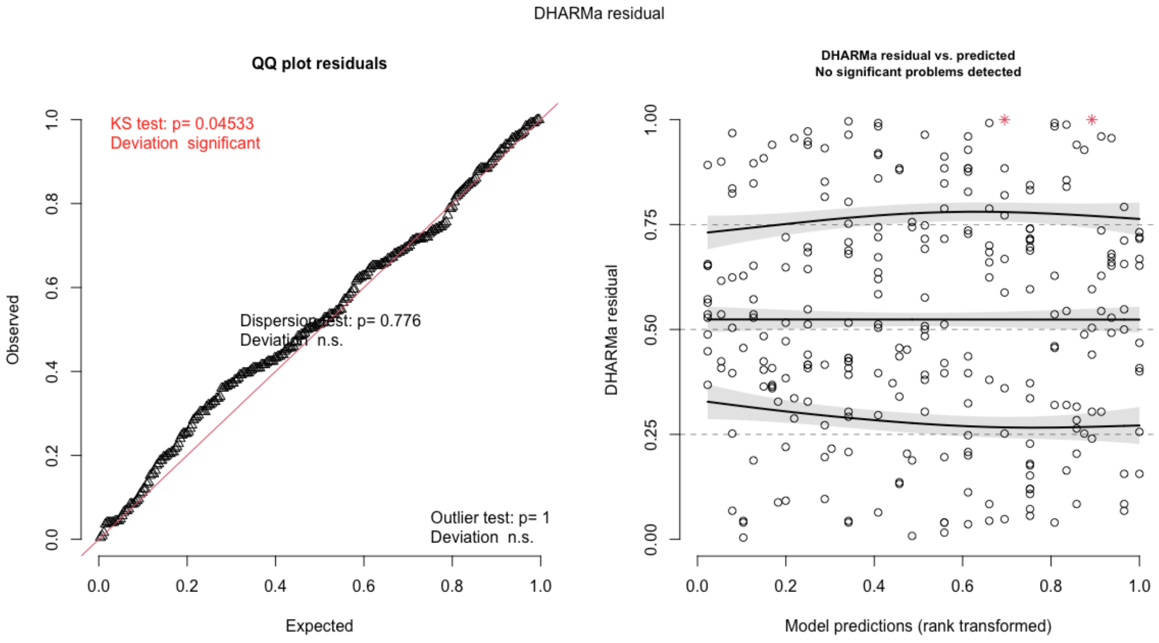

Figure S6. Pollinators contribution to nestedness

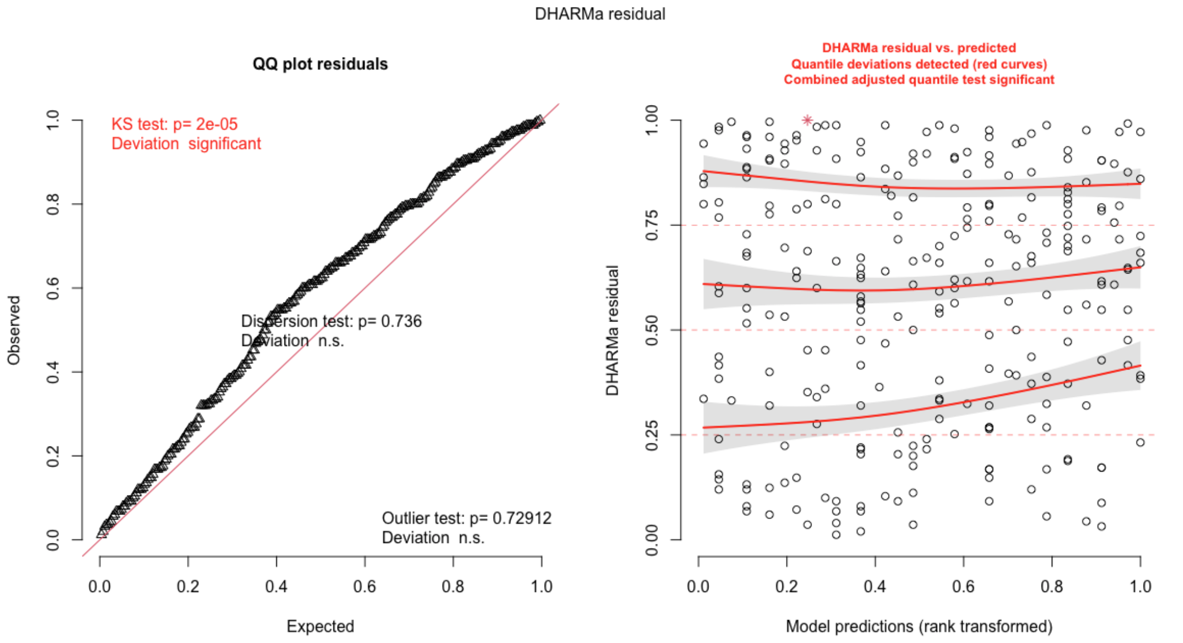

Figure S7. Pollinators species strength

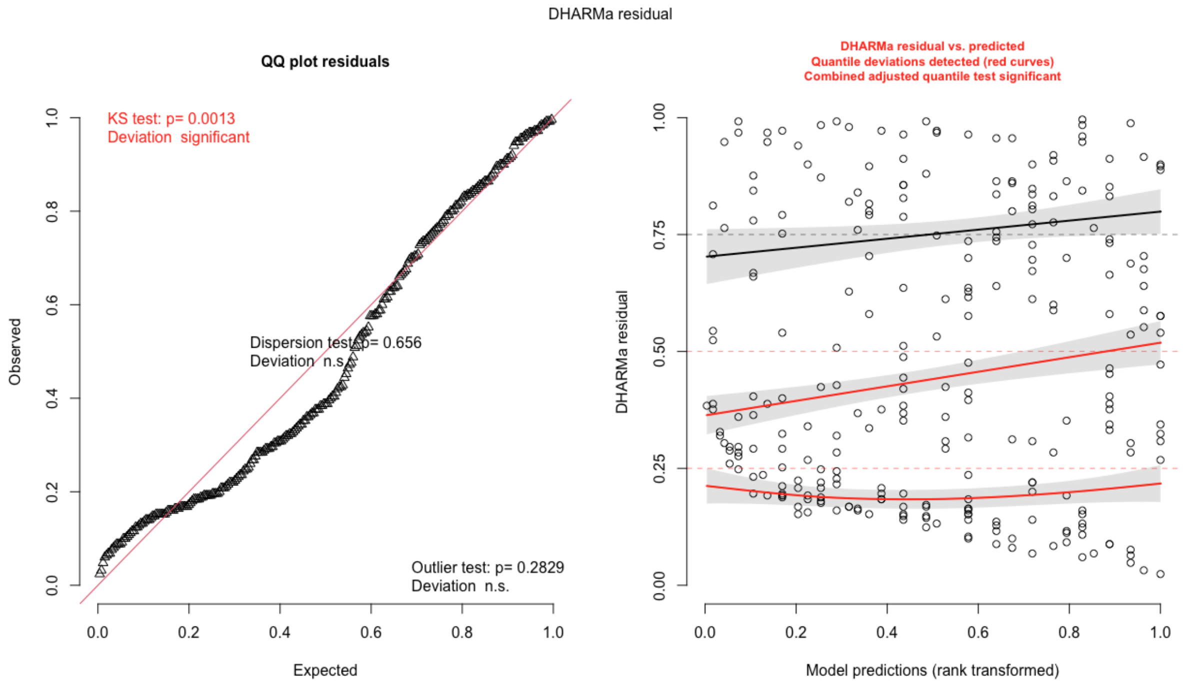

Figure S8. Pollinators specialisation degree (*d'*)

Table S4. Species-level metrics vegetation types pairwise comparisons. Pos-hoc estimated marginal means (*emmeans*) test, applying *holm* correction for multiple testing and Kenward–Roger approximation for degrees of freedom.

Vegetation type pairwise comparison	Normalised degree			Species contribution to nestedness			Species strength			Specialization degree (*d´*)		
	Estimate	SE	Estimate	Estimate	SE	*p* value	Estimate	SE	*p* value	Estimate	SE	*p* value
a) Plants												
Coastal – Coconut	−0.30801	0.361	1	1.2332	0.505	0.1439	−0.3122	0.301	1	-0.1189	0.123	1
Coastal – Garden	0.19524	0.241	1	0.0821	0.338	1	−0.2303	0.202	1	-0.0211	0.082	1
Coastal – Glacis	0.2027	0.209	1	0.1347	0.287	1	−0.2894	0.175	1	-0.1084	0.073	1
Coastal – Mixed	−0.06680	0.242	1	1.2957	0.332	**0.0019**	−0.2240	0.202	1	-0.0262	0.083	1
Coastal – Native	0.01494	0.216	1	−0.0433	0.291	1	−0.4036	0.18	0.4044	-0.0414	0.076	1
Coconut – Garden	0.50326	0.323	1	−1.1511	0.447	0.112	0.082	0.27	1	0.0978	0.115	1
Coconut – Glacis	0.51072	0.332	1	−1.0985	0.46	0.1483	0.0229	0.278	1	0.0105	0.116	1
Coconut – Mixed	0.24121	0.335	1	0.0625	0.468	1	0.0883	0.28	1	0.0926	0.119	1
Coconut – Native	0.32296	0.346	1	−1.2766	0.487	0.1074	−0.0914	0.289	1	0.0774	0.12	1
Garden – Glacis	0.00746	0.179	1	0.0526	0.255	1	−0.0591	0.149	1	-0.0873	0.065	1
Garden – Mixed	−0.26205	0.198	1	1.2136	0.284	**0.0005**	0.0063	0.165	1	-0.0052	0.074	1
Garden – Native	−0.18030	0.202	1	−0.1254	0.293	1	−0.1733	0.168	1	-0.0204	0.072	1
Glacis – Mixed	−0.26950	0.189	1	1.161	0.271	**0.0005**	0.0654	0.157	1	0.0822	0.071	1
Glacis – Native	−0.18776	0.182	1	−0.1780	0.261	1	−0.1142	0.152	1	0.067	0.067	1
Mixed – Native	0.08175	0.196	1	−1.3390	0.288	**0.0001**	−0.1796	0.163	1	-0.0152	0.073	1

b) Pollinators 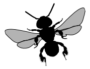

Coastal – Coconut	−0.09835	0.0338	**0.0439**	0.4671	0.277	0.7897	-0.0769	0.47	1	0.1175	0.077	0.568
Coastal – Garden	0.02127	0.0251	1	0.0459	0.206	1	-0.6582	0.355	0.8495	0.0859	0.051	0.5715
Coastal – Glacis	0.03382	0.0206	0.9193	−0.1339	0.168	1	-0.7801	0.298	0.1408	-0.0958	0.043	0.2425
Coastal – Mixed	0.00993	0.0226	1	0.6222	0.184	**0.0109**	-0.6001	0.33	0.8495	-0.043	0.047	1
Coastal – Native	−0.00376	0.0191	1	−0.2106	0.155	1	-0.2708	0.283	1	-0.106	0.04	1
Coconut – Garden	0.11962	0.03	**0.0013**	−0.4212	0.246	0.7897	-0.5813	0.435	1	-0.0317	0.062	1
Coconut – Glacis	0.13218	0.0299	**0.0002**	−0.6010	0.245	0.1478	-0.7032	0.431	1	-0.2133	0.062	**0.0088**
Coconut – Mixed	0.10829	0.0309	**0.0073**	0.1552	0.255	1	-0.5233	0.446	1	-0.1605	0.065	0.1631
Coconut – Native	0.09459	0.0313	**0.0339**	−0.6776	0.257	0.0995	-0.1939	0.44	1	-0.1282	0.063	0.3440
Garden – Glacis	0.01256	0.0194	1	−0.1798	0.16	1	-0.1219	0.285	1	-0.1817	0.041	**0.0003**
Garden – Mixed	−0.01133	0.0228	1	0.5764	0.187	**0.027**	0.058	0.33	1	-0.1289	0.048	0.0938
Garden – Native	−0.02503	0.0211	1	−0.2564	0.174	0.9931	0.3874	0.293	1	-0.0965	0.043	0.2425
Glacis – Mixed	−0.02389	0.02	1	0.7562	0.164	**<0.0001**	0.18	0.296	1	0.0528	0.043	1
Glacis – Native	−0.03759	0.0174	0.3143	−0.0766	0.143	1	0.5093	0.25	0.5947	0.0851	0.036	0.2147
Mixed – Native	−0.01370	0.0196	1	−0.8328	0.161	**<0.0001**	0.3294	0.289	1	0.0324	0.042	1

Table S5. GLMM statistical model results for plant species-level metrics

Response variable	Fixed efect	Estimate	SE	p value	Condt. R^2	Marginal R^2	95% CI
Plants: $N_{obs} = 142;\ N_{species} = 41$							
Normalized degree	Native * Invasion level	−0.01010	0.00419	**0.0176**	0.508	0.065	−0.01826 – −0.00203
	Non-native * Invasion level	0.00683	0.00393	0.0852.			−0.00099 – 0.01428
	Flower abundance	0.01231	0.01136	0.2811			−0.00899 – 0.03484
Contribution to nestedness	Native * Invasion level	−0.01144	0.00607	0.0618.	0.414	0.207	−0.02328 – 0.00013
	Non-native * Invasion level	0.00383	0.00567	0.5003			−0.00690 – 0.01482
	Flower abundance	−0.04140	0.01652	**0.0137**			−0.07219 – −0.00838
Species strength	Native * Invasion level	−0.00876	0.00348	**0.0131**	0.468	0.083	−0.01569 – −0.00219
	Non-native * Invasion level	0.00403	0.00321	0.2125			−0.00197 – 0.01101
	Flower abundance	0.0017	0.00915	0.8534			−0.01755 – 0.01887
Specialization degree (d')	Native * Invasion level	1.58E-03	1.58E-03	0.319	0.257	0.057	-0.002 to 0.006
	Non-native * Invasion level	8.19E-04	1.44E-03	0.569			-0.003 to 0.005
	Flower abundance	3.70E-03	4.06E-03	0.365			-0.006 to 0.015

Table S6. GLMM statistical model results for pollinator species-level metrics

Response variable	Fixed effect	Excluding undetermined pollinators: Estimate	SE	*p* value	Condt. R^2	Marginal R^2	95% CI	All pollinators: Estimate	SE	*p* value	Condt. R^2	Marginal R^2	95% CI
Pollinators: $N_{obs} = 265;\ N_{species} = 54$								Pollinators: $N_{obs} = 307;\ N_{taxa} = 75$					
Normalised degree	Native * Invasion level	0.000632	0.000472	0.182	0.455	0.086	−0.000282 – 0.001542	0.000617	0.000452	0.1741	0.442	0.1	−0.000256 – 0.001492
	Undetermined * invasion level	NA	NA	NA			NA	0.000724	0.000765	0.3442			−0.000737 – 0.002219
	Non-native * Invasion level	−0.000746	0.000449	0.0976			−0.001633 – 0.000109	−0.000720	0.000424	0.0911			−0.001567 – 0.000085
	Flower abundance	0.001885	0.00129	0.1461			−0.000575 – 0.004379	0.001328	0.001186	0.2641			−0.000921 – 0.003650
Contribution to nestedness	Native * Invasion level	0.00229	0.00387	0.5541	0.278	0.154	−0.00523 – 0.00973	0.00209	0.00381	0.5844	0.293	0.16	−0.00531 – 0.00942
	Undetermined * invasion level	NA	NA	NA			NA	0.00727	0.00618	0.2403			−0.00489 – 0.01907
	Non-native * Invasion level	−0.00360	0.00371	0.3337			−0.01054 – 0.00409	−0.00287	0.0036	0.4254			−0.00963 – 0.00448
	Flower abundance	−0.02531	0.01055	**0.0177**			−0.04680 – −0.00567	−0.02154	0.00992	**0.0309**			−0.04140 – −0.00303
Species strength	Native * Invasion level	0.00409	0.00701	0.5597	0.436	0.04	−0.00942 – 0.01768	-4.143e-01	4.953e-01	0.404	0.393	0.038	-0009 to 0.018
	Undetermined * invasion level	NA	NA	NA			NA	-1.445e-01	7.326e-01	0.843			-0.023 to 0.023
	Non-native * Invasion level	−0.00295	0.00656	0.6529			−0.01562 – 0.00974	-2.115e-03	6.464e-03	0.744			-0.015 to 0.010
	Flower abundance	−0.01924	0.01759	0.2754			−0.05320 – 0.01480	-1.839e-02	2.590e+02	0.273			-0.0.51 to 0.018
	Native * Invasion level	0.00104	0.00101	0.303			-0.003 to 0.001	1.045e-01	5.703e-02	0.069*			-0.003 to 0.001

Specialization degree (*d'*)	Undetermined * invasion level	NA	NA	NA	0.184	0.117		2.426e-01	9.145e-02	**0.008**	0.188	0.121	-0.006 to 0.001
	Non-native * Invasion level	0.00282	0.000957	**0.004**			0.001 to 0.005	2.928e-03	9.483e-04	**0.002**			0.001 to 0.005
	Flower abundance	0.00129	0.00254	0.612			-0.004 to 0.006	2.421e-03	2.431e-03	0.32023			-0.002 to 0.007

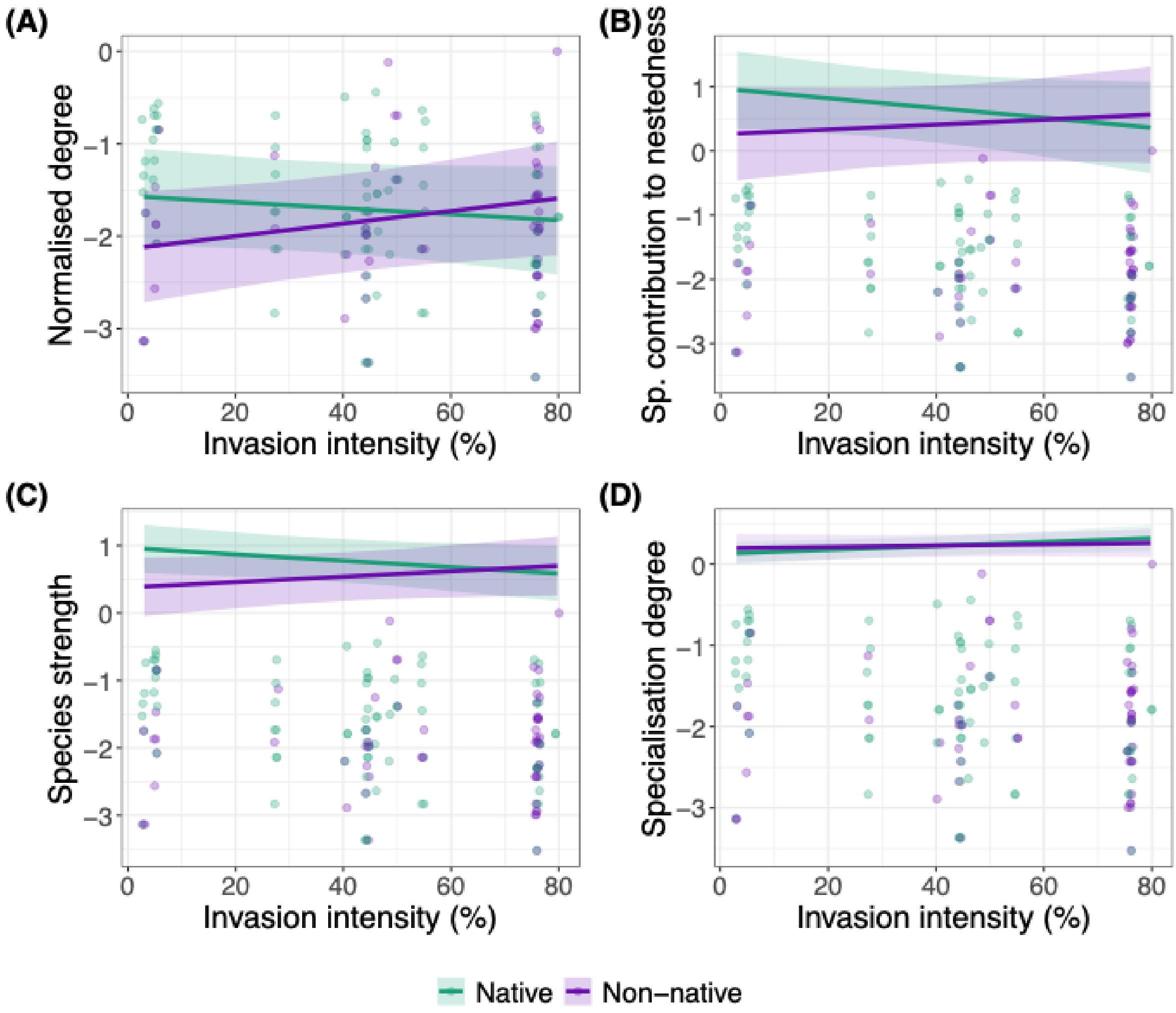

Figure S9. Predicted relationships between invasion intensity and plant species-level metrics for native and non-native plants. Solid lines show the predicted values from the generalized linear mixed models across the observed invasion gradient, with shaded areas representing 95% confidence intervals. Points represent individual observations. Panels show **A)** normalized degree, **B)** species contribution to nestedness, **C)** species strength, and **D)** specialization degree (*d′*).

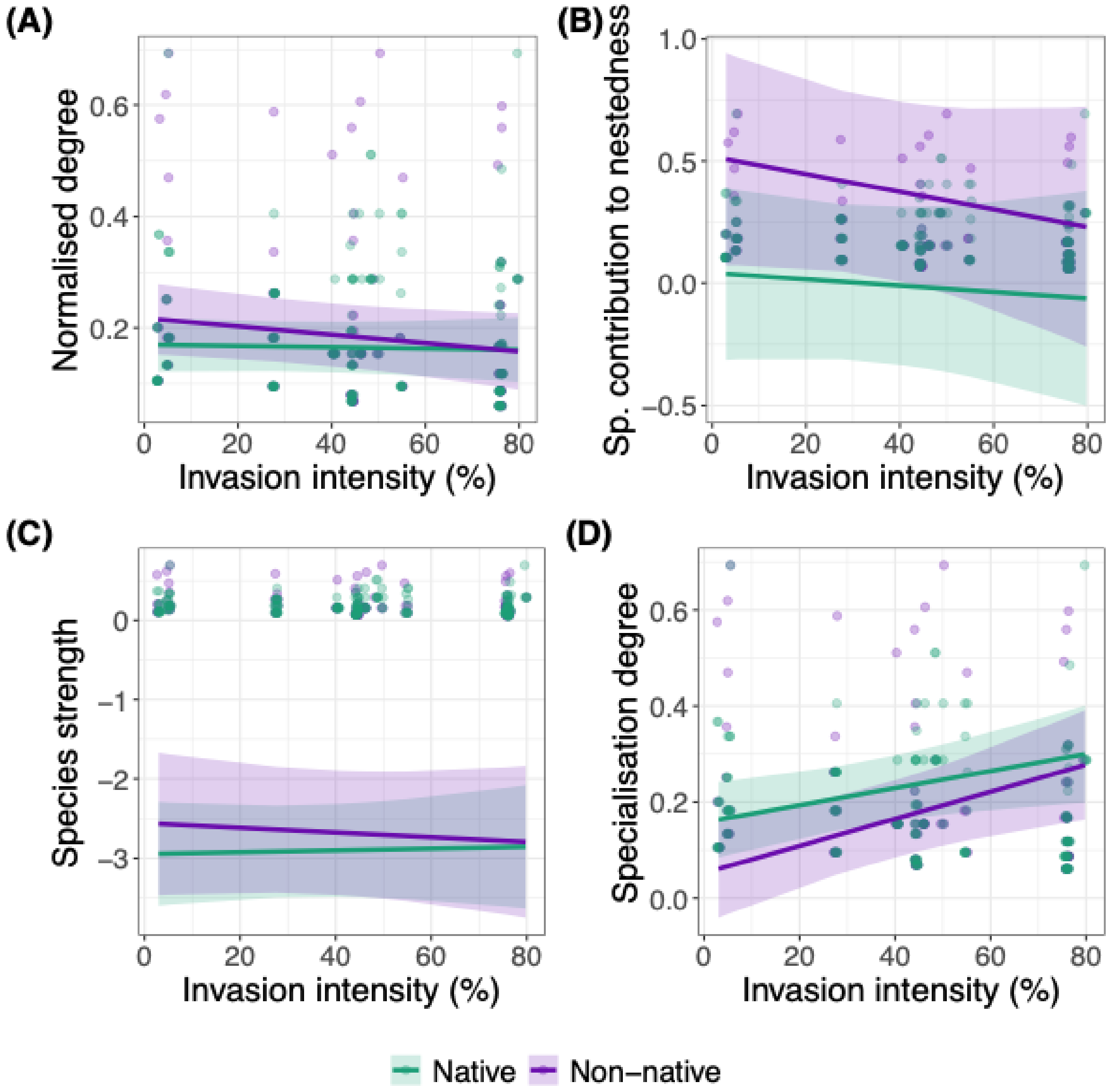

Figure S10. Predicted relationships between invasion intensity and pollinator species-level metrics for native and non-native pollinators. Solid lines show the predicted values from the generalized linear mixed models across the observed invasion gradient, with shaded areas representing 95% confidence intervals. Points represent individual observations. Panels show **A)** normalized degree, **B)** species contribution to nestedness, **C)** species strength, and **D)** specialization degree (d').
